# Clinical implementation of artificial-intelligence-assisted detection of breast cancer metastases in sentinel lymph nodes: the CONFIDENT-B single-center, non-randomized clinical trial

**DOI:** 10.1038/s43018-024-00788-z

**Published:** 2024-06-27

**Authors:** C. van Dooijeweert, R. N. Flach, N. D. ter Hoeve, C. P. H. Vreuls, R. Goldschmeding, J. E. Freund, P. Pham, T. Q. Nguyen, E. van der Wall, G. W. J. Frederix, N. Stathonikos, P. J. van Diest

**Affiliations:** 1 Department of Pathology, University Medical Center Utrecht, Utrecht, The Netherlands; 2 Department of Medical Oncology, University Medical Center Utrecht, Utrecht, The Netherlands; 3 Department of Epidemiology and Health Economics, Julius Center for Health Sciences and Primary Care, University Medical Center Utrecht, Utrecht, The Netherlands

**Keywords:** Cancer screening, Pathology, Machine learning, Cancer imaging, Breast cancer

## Abstract

Pathologists’ assessment of sentinel lymph nodes (SNs) for breast cancer (BC) metastases is a treatment-guiding yet labor-intensive and costly task because of the performance of immunohistochemistry (IHC) in morphologically negative cases. This non-randomized, single-center clinical trial (International Standard Randomized Controlled Trial Number:14323711) assessed the efficacy of an artificial intelligence (AI)-assisted workflow for detecting BC metastases in SNs while maintaining diagnostic safety standards. From September 2022 to May 2023, 190 SN specimens were consecutively enrolled and allocated biweekly to the intervention arm (*n* = 100) or control arm (*n* = 90). In both arms, digital whole-slide images of hematoxylin–eosin sections of SN specimens were assessed by an expert pathologist, who was assisted by the ‘Metastasis Detection’ app (Visiopharm) in the intervention arm. Our primary endpoint showed a significantly reduced adjusted relative risk of IHC use (0.680, 95% confidence interval: 0.347–0.878) for AI-assisted pathologists, with subsequent cost savings of ~3,000 €. Secondary endpoints showed significant time reductions and up to 30% improved sensitivity for AI-assisted pathologists. This trial demonstrates the safety and potential for cost and time savings of AI assistance.

## Abstract (web summary)

Van Dooijeweert et al. conducted a prospective study on the clinical implementation of artificial-intelligence-assisted detection of sentinel lymph node metastasis in persons with breast cancer and report on its effects, including on time and cost.

## Main

With an incidence of 2.3 million in 2020, breast cancer remains the most common type of cancer in women worldwide^1^. In the Netherlands, approximately 18,000 women and more than 100 men are diagnosed with breast cancer annually, translating into the development of breast cancer during life in about one in every seven women^2^. An important prognostic factor in breast cancer is the presence of (axillary) lymph node metastases^3^. Sentinel lymph nodes (SNs) are the first lymph nodes to drain lymphatic flow from the tumor; thus, the SN status predicts the likelihood of further axillary lymph node metastases, without the need for removing all (axillary) lymph nodes, thereby preventing major morbidity. The SN procedure is, therefore, performed in persons with breast cancer in whom diagnostic imaging is negative for involved axillary lymph nodes, which is the case in the majority of persons with breast cancer^3,4^. The SN procedure itself entails a combination of intratumor injections with radiocolloid and a perioperative injection of patent blue to detect and resect the SN(s)^3,4^. The presence of metastases in the SNs is strongly associated with worse survival^5–8^ and consequently guides treatment according to the size of the metastases (that is, macrometastases (≥2 mm), micrometastases (<2 mm) or isolated tumor cells (ITCs; single tumor cells or tumor cell clusters with a maximum diameter of ≤0.2 mm and a maximum number of 200 cells per section))^3^. In general, persons with an SN containing (micro)metastases, without prior neoadjuvant treatment, require adjuvant treatment, whereas those with a negative SN or only ITCs do not^3,4^.

For pathologists, the assessment of these SNs is a tedious and labor-intensive task with a dichotomous answer: the presence or absence of SN metastases. Subsequently, the SN slides have to be assessed diligently so as to not miss small but clinically relevant metastases. Meanwhile, the overall yield is low because the majority of SNs do not contain metastases (approximately two thirds). At the University Medical Center (UMC) Utrecht (the Netherlands), pathologists digitally assess SN specimens on regular hematoxylin and eosin (HE)-stained slides on a computer screen on five slides per SN tissue block. The current Dutch breast cancer guidelines prescribe that, if no metastases are morphologically identified on the HE slides, additional immunohistochemistry (IHC) stains are performed to rule out the presence of (mainly isolated) tumor cells^3^. However, these stains lead to high additional costs. The aggregate costs of IHC stains and pathologists’ time may easily exceed reimbursement for the full specimen in the case of multiple or large SNs as they have to be processed into multiple blocks, resulting in a high number of stains.

Because of the importance of the SN status in clinical management, as well as the time-consuming, repetitive and costly diagnostic workflow of SN assessment, an artificial intelligence (AI)-assisted approach has great potential to improve the (dichotomous) diagnostic SN workflow. In the current era of digital pathology, the number of studies on AI has increased exponentially^9,10^. Currently, AI algorithms have already been developed for various tasks such as tumor detection, tumor subtyping, (tumor) biomarker evaluation and tumor grading^9,11^ and the first algorithms have obtained Conformité Européene in vitro diagnostic (CE-IVD) marking^12^. Yet, prospective studies on actual clinical implementation are lacking, sometimes even many years after promising publications^13^. Interestingly, some of these algorithms have been shown to be superior to pathologists under time constraints^10^. However, independently operating algorithms, without pathologist supervision, are not preferred and perhaps even undesirable in cancer diagnostics for many reasons, including the ethical and legal consequences in cases where the AI-generated diagnosis is incorrect. Moreover, rather than being mutually exclusive, human intelligence and AI complement each other and outperform either one alone, a concept known as ‘augmented intelligence’^14^.

**Fig. 1:**
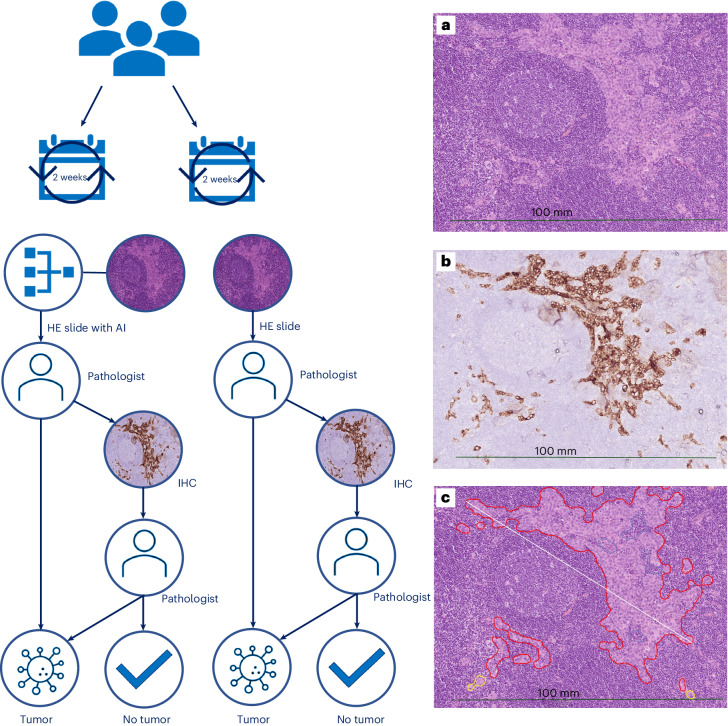
Study workflow of the CONFIDENT-B trial with example images of a single SN with different stains (regular HE, IHC and AI output). **a**–**c**, Example of an SN with micrometastases on the regular HE slide (**a**), on the slide stained by IHC (**b**) and on the regular HE slide with the output of the Metastasis Detection app by Visiopharm (**c**). Outlines in yellow (low probability) and red (high probability) show potential metastases. Blue outlines concern excluded regions within annotations. The example images were derived from one of the cases in the control arm of the CONFIDENT-B trial. In this case, the micrometastases were missed by the unassisted pathologist on HE (**a**), after which IHC was performed (**b**). Afterward, for the assessment of standalone AI performance, we ran the algorithm (**c**), resulting in these three images of the same SN.

In this single-center trial, we prospectively investigated the relative risk (RR) of IHC use per detected case of SN metastasis of an AI-assisted clinical workflow for the detection of SN metastases in breast cancer, a tumor localization task for which the reference standard was our daily practice of pathologist supervision with or without IHC (Fig. 1). Our main objective was to examine to which extent the AI-assisted workflow could reduce the material and personnel resources spent on IHC stains, while maintaining diagnostic safety standards.

## Results

A total of 190 SN specimens from 182 participants were included, of which 100 were included in the AI arm (52.6%) and 90 were included in the control arm (47.4%). Characteristics of the included SN specimens are presented in Table 1. The mean age of participants was 57.2 ± 11.6 years (mean ± s.d.) and all participants but one were of female sex. Over 40% of participants received neoadjuvant therapy, nearly always consisting of at least chemotherapy, and up to almost 40% of this group also underwent an MARI procedure (marking the axillary lymph node with radioactive iodine (^125^I) seeds)^15^ because of a proven tumor-positive axillary lymph node before the start of neoadjuvant therapy. In many cases (17 of 30), the MARI node was also the SN.

**Table 1:** Participant, tumor and pathologist characteristics of the 190 included SN specimens

	Total (*n* = 190)	AI arm (*n* = 100)	Control arm (*n* = 90)	*P* value
**Participants**				
Number of unique participants^a^	182 (95.8%)	97 (97.0%)	85 (94.4%)	0.360
Age in years per participant, mean (s.d.)	57.2 (11.6)	58.0 (11.1)	56.3 (12.1)	0.309
Neoadjuvant treatment	77 (42.3%)	39 (40.2%)	38 (44.7%)	0.540
MARI procedure	30 (16.5%)	14 (14.4%)	16 (18.8%)	0.709
**Hospital and pathologists per SN specimen**				
Hospital				
UMC Utrecht	28 (14.7%)	14 (14.0%)	14 (15.6%)	0.763
Alexander Monro Hospital	162 (85.3%)	86 (86.0%)	76 (84.4%)	
Expert breast pathologist				
Pathologist A	99 (52.1%)	55 (55.0%)	44 (48.9%)	0.122
Pathologist B	49 (25.8%)	25 (25.0%)	24 (26.7%)	
Pathologist C	24 (12.6%)	15 (15.0%)	9 (10.0%)	
Pathologists D and E	18 (9.5%)	5 (5.0%)	13 (14.4%)	
**Unilateral SN specimens**				
Number of SNs, mean (s.d.)	1.65 (1.12)	1.70 (1.30)	1.59 (0.09)	0.704
Number of blocks, mean (s.d.)	1.81 (1.04)	1.89 (1.02)	1.72 (0.11)	0.138
SN status				
Absence of SN metastases	131 (68.9%)	75 (75.0%)	56 (62.2%)	0.036
ITCs	18 (9.5%)	11 (11.0%)	7 (7.8%)	
Micrometastases	24 (12.6%)	10 (10%)	14 (15.6%)	
Macrometastases	17 (8.9%)	4 (4.0%)	13 (14.4%)	
Neoadjuvant treatment	80 (42.1%)	41 (41.0%)%)	39 (43.3%)	0.745
MARI procedure	30 (15.7%)	14 (14.1%)	16 (17.7%)	0.525
Unilateral tumor characteristics				
(y)pT stage				
(y)pT0	23 (12.1%)	13 (13.0%)	10 (11.1%)	0.621
(y)pTis	27 (14.2%)	14 (14.0%)	13 (14.4%)	
(y)pT1	89 (46.8%)	49 (49.0%)	40 (44.9%)	
(y)pT2	42 (22.1%)	18 (18.0%)	24 (26.7%)	
(y)pT3–4	9 (4.7%)	6 (6.0%)	3 (3.3%)	
Histologic subtype				
Invasive carcinoma NST	113 (59.5%)	56 (56.0%)	57 (63.3%)	0.754
Invasive lobular carcinoma	20 (10.5%)	12 (12.0%)	8 (8.9%)	
Invasive ductulolobular carcinoma	17 (8.9%)	11 (11.0%)	6 (6.7%)	
Invasive carcinoma any other type	12 (6.3%)	6 (6.0%)	6 (6.7%)	
Carcinoma in situ	28 (14.7%)	15 (15.0%)	13 (14.4%)	
Histologic grade^b^				
Grade I	38 (20.0%)	19 (19.0%)	19 (21.1%)	0.414
Grade II	89 (46.8%)	50 (50.0%)	39 (43.3%)	
Grade III	61 (32.1%)	31 (31.0%)	30 (33.4%)	
Grade not applicable^c^	2 (1.1%)	0 (0.0%)	2 (2.2%)	
Lymphovascular invasion				
Present	24 (13.2%)	13 (13.0%)	12 (13.3%)	0.664
Absent	144 (75.8%)	74 (74.0%)	70 (77.8%)	
Unknown	21 (11.1%)	13 (13.0%)	8 (8.9%)	
ER status				
Positive	136 (71.6%)	76 (76.0%)	60 (66.7%)	0.160
Negative	30 (15.8%)	11 (11.0%)	19 (21.1%)	
Unknown	24 (12.6%)	13 (13.0%)	11 (12.2%)	
PR status				
Positive	118 (62.1%)	65 (65.0%)	53 (58.9%)	0.550
Negative	48 (25.3%)	22 (22.0%)	26 (28.9%)	
Unknown	24 (12.6%)	13 (13.0%)	11 (12.2%)	
HER2 status				
Positive	29 (15.3%)	15 (15.0%)	14 (15.6%)	0.992
Negative	138 (72.6%)	73 (73.0%)	65 (72.2%)	
Unknown	23 (12.1%)	12 (12.0%)	11 (12.2%)	

**Abbreviations**: (y)pT, tumor stage after neoadjuvant therapy; NST, no specific type; ER, estrogen receptor; PR, progesterone receptor; HER2, human epidermal growth factor receptor 2.

^a^Eight participants had bilateral DCIS or breast cancer.

^b^In the case of a complete response after neoadjuvant therapy, the histologic grade was taken from the invasive component on the biopsy; in the absence of an invasive component, the histologic grade was taken from the DCIS component.

^c^Lobular carcinoma in situ was not graded.

The majority of SN specimens were derived from the Alexander Monro hospital (85.3%) and about one half of all specimens were assessed by pathologist A (52.1%). Pathologists D and E were grouped together as they were included in the study for only a short period of time. On average, pathologists assessed 1.65 ± 1.12 SNs (mean ± s.d.) and approximately nine slides per SN specimen (1.81 blocks × 5).

Overall, 59 SN specimens contained metastases (31.1%), consisting of a similar proportion of ITCs (*n* = 18) and macrometastases (*n* = 17), while micrometastases (*n* = 24) were most common. In contrast to all other (tumor) characteristics, SN status significantly differed between both study arms (Table 1). In the AI arm, there were significantly more negative cases; of the detected metastases, the majority consisted of ITCs, as opposed to a larger proportion of micrometastases and macrometastases in the control arm.

### IHC use

Overall, IHC was used in 154 SN specimens (*n* = 1,335 stains), which detected SN metastases in 25 SN specimens at a total cost of 33,275 € (an estimated 25 € per stain).

IHC use per detected case of SN metastasis is summarized in Table 2. IHC was needed to detect ITCs in most cases, micrometastases in some cases and macrometastases in rare cases. After correcting for metastasis size, the adjusted RR (aRR) for IHC use per detected case of SN metastasis showed a significantly lower risk of IHC use for AI-assisted pathologists (aRR = 0.680, 95% confidence interval (CI): 0.347–0.878). As can be seen in the stratified analysis (Table 2), the use of AI assistance mainly prevented IHC use for the detection of micrometastases (RR = 0.400, 95% CI: 0.069–1.264).

**Table 2:** IHC use for the detection of cases of SN metastases

	Total (*n* = 59)	AI arm (*n* = 25)	Control arm (*n* = 34)	*P* value	RR of IHC use per case of SN metastasis (95% CI)	aRR of IHC use per case of SN metastasis (95% CI)*	*P* value*
IHC use for detection of SN metastases	25 (42.4%)	10 (40.0%)	15 (44.1%)	0.752	0.907 (0.467–1.654)	0.680 (0.347–0.878)	0.020
IHC use for detection per type of SN metastasis							
ITCs	(*n* = 18)	(*n* = 11)	(*n* = 7)				
Number of cases with ITCs for which IHC stains were used	15 (83.3%)	8 (72.7%)	7 (100%)	0.130	NA**	−	−
Micrometastases	(*n* = 24)	(*n* = 10)	(*n* = 14)				
Number of cases micrometastases for which IHC stains were used	9 (37.5%)	2 (20.0%)	7 (50.0%)	0.210	0.400 (0.069–1.264)	−	−
Macrometastases	(*n* = 17)	(*n* = 4)	(*n* = 13)				
Number of cases with macrometastases for which IHC stains were used	1 (5.9%)	0 (0.0%)	1 (7.7%)	1.000	NA**	−	−

*The RR was corrected for metastasis size, 95% CI by bootstrapping.**NA, not applicable, due to 0 and 100% values.

The number of IHC stains used per detected case of SN metastasis was lower in the AI arm (85 stains versus 150 stains), resulting in an average expenditure of 85 € per detected case of SN metastasis in the AI arm versus 110 € in the control arm. In addition, AI assistance reduced the cost per detected case for all types of SN metastases (ITCs, 170 € versus 321 €; micrometastases, 25 € versus 89 €; macrometastases, 0 € versus 19 €).

Of all identified cases of ITCs (*n* = 18), finding these cells had possible clinical consequences in only five participants (27.8%) as they had received neoadjuvant treatment. Of these five cases, the algorithm, either assisted by a pathologist (*n* = 2 of 3) or standalone (*n* = 2 of 2), identified four (80%).

### Workflow improvement

Overall, 27 time measurements were performed on pathologists A and B, 15 in the AI arm (pathologist A, *n* = 3; pathologist B, *n* = 12) and 12 in the control arm (pathologist A, *n* = 1; pathologist B, *n* = 11). Pathologists in the AI arm spent significantly less time on their assessment of the HE slides (3 min 41 s) than pathologists in the control arm (6 min 4 s) (*P* = 0.028). Importantly, this was despite the fact that, in the AI arm, significantly more slides (on average, 11.7 slides versus 7.5 slides) were assessed during the time measurements (*P* = 0.041). In addition, not taken into account in these time measurements is that the assessment of IHC-stained slides also takes pathologists approximately 1 min per SN specimen (scanning for ‘brown cells’). In the AI arm, the assessment of IHC-stained slides was performed in a significantly lower proportion of SNs, thereby saving even more of the pathologists’ time.

### Pathologist performance

The pathologists’ performance for both arms is summarized in Table 3. The overall sensitivity of the unassisted pathologists was 55.8%, while their negative predictive value (NPV) was 78.9%. The overall sensitivity of AI-assisted pathologists was 60%, with an NPV of 88.2%. Again, when stratified for type of metastasis, sensitivity with AI assistance was gained for all types of metastases but most for the detection of micrometastases and ITCs (+30% and +27.3%, respectively).

**Table 3:** Pathologist performance

	Pathologist on HE slides (+)	Pathologist on HE slides (−)	Sensitivity (95% CI)	NPV (95% CI)
Control arm (unassisted pathologist)				
Metastases (+)	19	15	55.9% (37.9–72.8%)	
ITCs	0	7	0.0% (0.0–41.0%)	
Micrometastases	7	7	50.0% (23.0–77.0%)	
Macrometastases	12	1	92.3% (64.0–99.8%)	
Absence of metastases (−)	0	56	-	78.9% (67.6–87.7%)
Intervention arm (AI-assisted pathologist)				
Metastases (+)	15	10	60.0% (38.7–78.9%)	
ITCs	3	8	27.3% (6.0–61.0%)	
Micrometastases	8	2	80.0% (44.4–97.5%)	
Macrometastases	4	0	100.0% (39.8–100.0%)	
Absence of metastases (−)	0	75	−	88.2% (79.4–94.2%)

The pathologists’ performance was derived from the SN status based on the pathologists’ conclusion with or without the use of IHC.

Overall, four of five participating pathologists used the algorithm during the trial. All four AI-using pathologists finished the user experience survey (Extended Data Table 1). They strongly agreed that the algorithm was easy to use (score 4.75 out of 5), that it saved them time while reviewing SNs (score 4.5 out of 5) and that they felt confident using it in their diagnostics (score 4.75 out of 5). Furthermore, they (strongly) agreed that the algorithm made their work more enjoyable (score 4.25 out of 5) and that they wanted to continue using the algorithm to evaluate SNs in breast cancer (score 4.5 out of 5). Three of four pathologists agreed that they trusted the output of the algorithm (score 4 out of 5), while the fourth answered with a neutral score of 3.

### Algorithm performance

**Extended Data Fig. 1:**
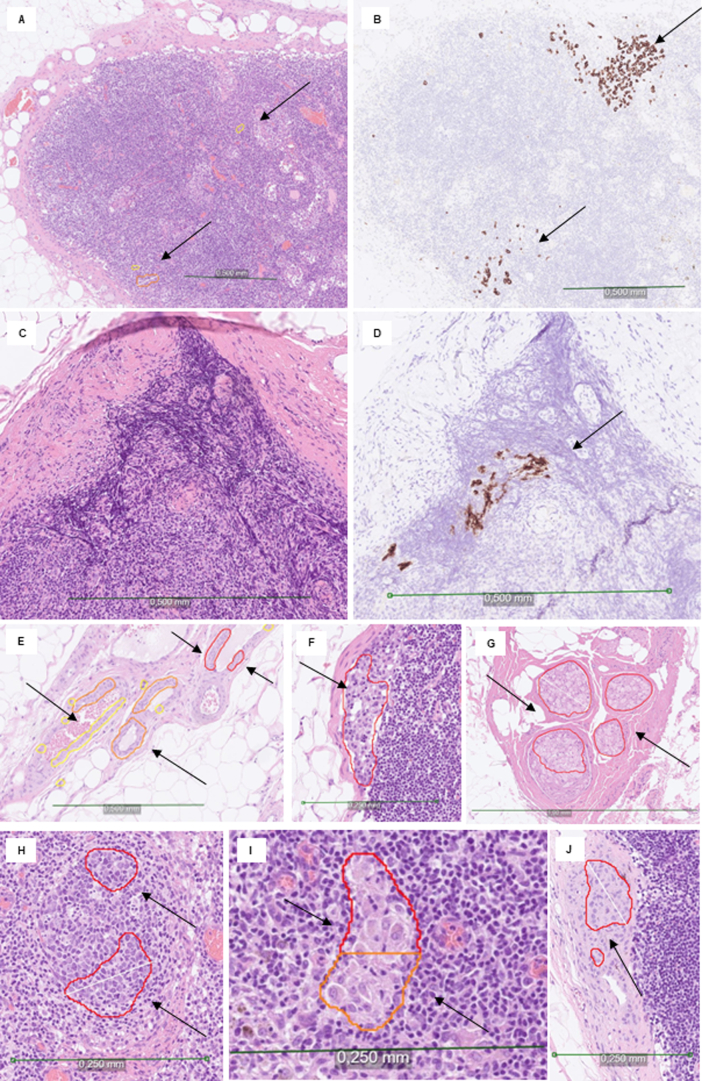
Example images of two cases of initially undetected micro-metastases (false negatives) in the AI-assisted arm (A-B & C-D) and false positive alerts of the Metastasis Detection App by Visiopharm (E-J). **a/b**: Sentinel node with micro-metastases on the regular HE-slide partly highlighted (in yellow and orange) by the algorithm, that was overlooked by the AI-assisted pathologist on the HE slide (A) and the detected micro-metastasis on the IHC-stained slide (B). **c/d**: Sentinel node with micro-metastases located in a heavily cauterized area on the HE-section (C), which therefore could only be detected in the IHC-section (D). **e-j**: False positive alerts by AI: blood vessels highlighted in red, yellow and orange (A), sinus histiocytosis highlighted in red (B), nerves highlighted in red (C), a follicle center highlighted in red (D), a pigment laden macrophage highlighted in red and orange (E) and a capsular naevus (F) in red. The example images are snapshots derived from whole slide images of sentinel lymph nodes included in the CONFIDENT-B trial.

The standalone algorithm performance in both arms, as well as the overall performance, is summarized in Table 4. In contrast to a lower sensitivity for ITCs (44.4%), the algorithm picked up all 17 macrometastases and all but one of the micrometastases, resulting in a sensitivity of 95.8%. Although the AI-assisted pathologist missed two cases of micrometastases, the algorithm did actually outline these tumor cells in one case (in yellow and orange), yet this was overlooked by the pathologist for some reason (Extended Data Fig. 1). As for the other case of missed micrometastasis by both the algorithm and the AI-assisted pathologist, the tumor cells were located in a heavily cauterized area of the HE slide and, therefore, only visible on the IHC slides (serial section); accordingly, they would not have been picked up on this specific HE slide. For the control arm, the algorithm retrospectively picked up all micrometastases and macrometastases and nearly one half of the ITCs. Thus, it could have prevented a total number of 115 stains used in daily practice, indicating that, in a time span of 16 weeks, an expenditure of 2,875 € could have been prevented by the use of AI.

**Table 4:** Standalone algorithm performance

	Standalone AI on HE slides (+)	Standalone AI on HE slides (−)	Sensitivity (95% CI)
Control arm (analysis in retrospect)			
Metastases (+)			
ITCs	3	4	42.9% (9.9–81.6%)
Micrometastases	14	0	100.0% (76.8–100.0%)
Macrometastases	13	0	100.0% (75.3–100.0%)
Intervention arm			
Metastases (+)			
ITCs	5	6	45.5% (16.7–76.6%)
Micrometastases	9	1	90.0% (55.5–99.7%)
Macrometastases	4	0	100.0% (39.8–100.0%)
Overall			
Metastases (+)	48	11	81.4 (69.1–90.3%)
ITCs	8	10	44.4% (21.5–69.2%)
Micrometastases	23	1	95.8% (78.9–99.9%)
Macrometastases	17	0	100.0% (80.5–100%)

The standalone algorithm performance was derived from AI outlines checked by one of the researchers (C.v.D.) in consultation with one of the pathologists (P.J.v.D.) in cases of doubt.

### False-positive alerts

False-positive alerts by AI included mainly blood vessels, histiocytes, follicle centers, nerves and capsular naevi (Extended Data Fig. 1), which were easily recognizable as such for the pathologists. These false positives mainly occurred in the yellow (low suspicion) and orange (intermediate suspicion) classes. On average, pathologists reviewed 7.1, 1.7 and 1.7 yellow, orange and red annotations per slide, respectively. The average number of these false-positive alerts was slightly higher in SNs of participants who received neoadjuvant treatment (yellow, 10.0 versus 5.4; orange, 2.1 versus 1.5; red, 3.1 versus 0.9).

### Potential impact

In total, 16 laboratories from all regions in the Netherlands responded to our survey, which covers about one third of the pathology laboratories in the Netherlands (Table 5). Of these laboratories, the majority (81.3%) at the time evaluated the SN blocks on three levels, which is stated as the minimum number in the breast cancer guideline^3^. Yet, one laboratory stated that they only evaluated SN blocks at one level. Furthermore, in most laboratories (75%), IHC was performed before viewing the HE slides. This indicates that, although fewer stains were performed per block in most cases, evaluating the HE slides using the algorithm would save even more IHC stains.

**Table 5:** Survey among 16 pathology laboratories on their SN workflow in breast cancer

At how many levels do you examine the SN for tumor cells?	No. of labs (%)
1	1 (6.3%)
3	13 (81.3%)
5	2 (12.5%)
**When do you use IHC?**	
Always	12 (75.0%)
Always, unless macroscopically evident metastases	2 (12.5%)
When metastases are morphologically absent on HE slides	2 (12.5%)

In a future scenario where AI assistance is used in all cases and current safety standards are maintained (that is, IHC in all morphologically negative cases), potential cost savings per 100 unilateral SNs range from 1,500 to 3,500 €, depending on laboratory policy (that is, three or five slides per block and IHC up front or not) (Methods).

## Discussion

In this single-center prospective trial, real-time clinical implementation of AI assistance resulted in a significantly lower risk of IHC use per detected cases of SN metastasis (aRR = 0.680, 95% CI: 0.347–0.878). The use of AI assistance mainly prevented the use of IHC for the detection of micrometastases and reduced the cost of IHC use per detected case of SN metastasis for all types of metastases (that is, ITCs, micrometastases and macrometastases). In addition to preventing IHC use, thereby reducing costs, AI-assisted pathologists also spent significantly less time on their assessment of the HE slides of the SN specimens (3 min 41 s versus 6 min 4 s). In addition, the participating pathologists stated that AI was easy to use, that they felt confident using AI and that, in addition to saving them time, AI made their work more enjoyable. Furthermore, both the sensitivity and the NPVs of AI-assisted pathologists were higher for all types of SN metastases, yet again most strikingly for micrometastases (80% for AI-assisted pathologists versus 50% for unassisted pathologists).

Moreover, the standalone performance of AI showed excellent overall sensitivity for both micrometastases (95.8%) and macrometastases (100%), while being less sensitive for ITCs (44.4%). Furthermore, the single case of micrometastasis that was not detected or highlighted by AI could not have been prevented, as it was because of heavy cauterization of the specific area in that specific slide, which made it impossible to detect. Hence, it may be concluded that AI did not miss any micrometastases in this series. Although our research question was not whether the algorithm could perform independently, these findings do show the trustworthiness of the algorithm and that, in one of the cases where micrometastases were missed by the AI-assisted pathologist, the algorithm did highlight them (albeit partially and in yellow and orange). We assume that the AI-assisted pathologist missed the highlighted micrometastases because they did not review this annotation as it was in the yellow (low suspicion) and orange (intermediate suspicion) categories. This may be understandable because the yield of metastases in the yellow category is very low; nevertheless, this mistake indicates that all annotations by the algorithm, even the low-suspicion ones, need to be carefully reviewed. For this, the display of the Visiopharm app is not yet optimal because going from one annotation to the next may be time consuming when there are many yellow annotations. A display in three (yellow, orange and red) galleries within Sectra’s Picture Archiving and Communication System (PACS), as we previously achieved through full integration of our in-house mitoses algorithm^16^, will much better facilitate and even speed up annotation review and is a mandatory next step for routine clinical use.

Unexpectedly, the number (34 in the control arm versus 25 in the AI-assisted arm) and the type (ITC, micrometastases and macrometastases) of found metastases significantly differed between both study arms. This raised the question of whether metastases may have been missed, especially in the AI arm. However, because we performed IHC in all morphologically negative cases, this can be ruled out. By design, as in clinical practice, false-positive cases cannot exist as no confirmatory stains are performed when the pathologist (AI-assisted or unassisted) concludes that metastases are present on the HE slides. Therefore, the specificity and the positive predictive values (PPVs) were not presented. However, as shown by Challa et al. and as argued in the Methods, more false-positive diagnoses by a pathologist when using AI are highly unlikely. In addition, we detected significantly fewer metastases in the AI arm than in the control arm (75.0% versus 62.2%; Table 1). Nevertheless, as we are investigating tumor detection, the sensitivity and the NPVs are most important because metastases should not be missed.

Regarding current diagnostic safety standards, our survey clearly showed that these are not the same in all pathology laboratories. In contrast to our assessment of five levels per SN tissue block, most laboratories assessed three levels of HE slides per tissue block and one laboratory assessed only a single level per tissue block (against the current guideline). It is important to realize that IHC is performed on the total number of levels being assessed; hence, in the UMC Utrecht assessment of five levels per tissue block, and that these IHC slides still need to be assessed and quantified by pathologists, thus being subject to interpathologist variation. A good example is the central pathology review of almost 3,000 SN specimens from persons with early (favorable) primary breast cancer in the MIRROR study^7^, which included IHC stains in all negative cases. Here, the central pathology review resulted in a change in lymph node stage (N stage) in 24% of cases, which mainly consisted of upstaging^17^.

Although many promising AI pathology studies have been published^9–14^, sometimes even more than half a decade ago^13^, this has seemingly not yet resulted in clinical implementation and prospective studies. This may be because of a lack of a digital workflow in many laboratories; however, digital pathology is on the rise worldwide and has, for example, been introduced in about one half of the Dutch pathology laboratories. This prospective trial on the clinical implementation of AI in daily pathology practice investigated the added value of AI assistance while maintaining (and assessing) diagnostic accuracy and safety standards. By focusing on tangible savings, in both time and cost, we believe that this clinical trial template for tumor detection models may pave the way for broader implementation of such AI models in diagnostic pathology and help to build a business case for AI implementation. The latter is important because, in many countries, there is and will be no specific reimbursement for AI use in pathology. This is unfortunate and unjustified because optimal (AI-assisted) pathology will cost just a little bit more and will save much more money elsewhere^18^. Moreover, for pathology laboratories that are not yet fully digital, tangible potential cost savings from AI assistance may be an incentive or even a selling point to hospital management to support digitalization. As the market price of AI algorithms in digital pathology is not well defined (for example, the algorithm used here was part of a one-off package license to which future algorithms will be added (for 7 years); accordingly, the exact price we paid cannot be given), tangible cost savings from these kinds of prospective studies will determine what laboratories can and would be willing to spend on the purchase of these algorithms. However, informal consultations with several companies seem to suggest that a price of 1–3 € per image is deemed reasonable, easily outweighed by the cost savings of 25 € per omitted IHC stain.

By extrapolating the outcomes in the control arm to the total number of metastases in this trial, at an average number of nine slides per SN with 25 € per IHC stain, cost savings are estimated to be ~3,000 € (estimated cost of 36,450 € with no AI versus actual spendings of 33,275 € during the trial). Furthermore, the retrospective analysis of AI in the control arm showed that, within a time span of only 16 weeks, a similar amount (2,875 €) could have been saved. This shows that, by implementing AI in its current form while maintaining the safety net of IHC, substantial cost savings can already be reached within a relatively short time span of 32 weeks. However, as metastases are absent in two thirds of SN specimens, this is still were most money on IHC is spent. Whether it is safe and acceptable to forgo IHC stains in all AI-assisted morphologically negative cases is another discussion, which we elaborate on below.

In this regard, it is important to mention that micrometastases and macrometastases, according to current international guidelines, usually have therapeutic consequences for persons with (early) breast cancer, whereas ITCs in principle do not^3^. ITCs only have therapeutic consequences in persons who have had neoadjuvant therapy (42.3%), as these tumor cells are then considered residual disease^3^. We showed here that AI did not miss any macrometastases and micrometastases (with the exception of one unfortunate and unpreventable case of micrometastasis in a cauterized area) and also found almost half of all ITCs. We, therefore, propose to use AI assistance in all cases and to only use IHC in AI-assisted morphologically negative cases in persons who have received neoadjuvant treatment (‘personalized IHC use scenario’). Of course, this comes at a risk of potentially missing (relevant) micrometastases at some point along the way, because missing 0 of 24 micrometastases does not imply that AI (and the AI-assisted pathologist) would not miss 1 of 1,000 micrometastases. However, as mentioned above, it is important to realize that IHC itself does also not provide a diagnosis with 100% certainty. Cutting and assessing five HE-stained and five IHC-stained 4-µm sections per block still means that only 10% of the entire SN block is assessed. We believe that this minimalized risk of missing micrometastases according to the abovementioned policy outweighs the excessive costs of searching for ITCs in SNs of persons with breast cancer without any therapeutic consequences, especially in light of the current public debate on skyrocketing healthcare costs and limited resources.

To supplement the discussion about cost savings, estimations of cost savings for future scenarios ‘maintaining current safety standards’ versus ‘personalized IHC use’ were calculated in more detail for our own laboratory (five slides, using IHC staining when HE staining is morphologically negative) and for the two other most common laboratory practices (three slides, always using IHC staining; three slides, using IHC staining when HE staining is morphologically negative). These potential cost savings were calculated using parameters from this trial: 25 € per stain, an average of 1.81 tissue blocks per unilateral SN sample, proportions of negative SNs (68.9%) and SNs with specific types of metastases (ITCs, 9.5%; micrometastases, 12.6%; macrometastases, 9.0%) and the current and future proportions of IHC use extracted from the control arm and the overall sensitivity of AI. For the ‘maintaining current safety standards’ scenario, when keeping the current number of slides per tissue block (that is, three or five), potential cost savings per 100 unilateral SNs range from 1,500 to 3,500 €. In contrast, for the ‘personalized IHC use’ scenario, when maintaining the number of slides per tissue block (that is, three or five), cost savings per 100 unilateral SNs range from 7,500 to 12,500 €. Potential cost savings of individual pathology laboratories can be calculated by adjusting all relevant parameters in (Methods). Importantly, because an official market price and the associated costs for AI technology (for example, hardware and information technology experts) cannot be given at this point, these were not incorporated in the scenarios in (Methods). However, with these scenarios, laboratories will be able to calculate what they can and are willing to spend on the algorithm(s).

The few minutes of time saved do not immediately provide tangible cost savings. However, if multiple SNs need to be assessed in 1 day, time savings would become more tangible and eventually lead to a reduced workload. Furthermore, additional time would be saved as fewer IHC slides need to be assessed by pathologists. Moreover, the fact that pathologists mentioned that the algorithm was easy to use and that it made their work more enjoyable should also be an important incentive. Lastly, AI assistance reduces the workload of laboratory staff who have to cut and stain IHC slides and reduces the physical and digital storage space of these IHC slides. These factors all contribute to sustainable workforce deployment, which is desperately needed in an era of rising cancer diagnoses and an already existing global shortage of pathologists^19^.

Interestingly, the algorithm also helped pathologists to detect some relevant benign structures such as capsular naevi. Other false positive alerts included blood vessels, histiocytes, follicle centers and nerves, which were easily recognizable as such. These false positive alerts especially occurred in the yellow (low suspicion) and orange (intermediate suspicion) classes. Nevertheless, they highlight that there is still room for improvement of the algorithm, albeit not at the cost of sensitivity.

An important strength of this study was the prospective trial design, where AI assistance was directly used in diagnostic decision-making on all consecutive SN cases. Accordingly, the outcomes of this trial are generalizable for our laboratory. Because of the different workflows in other laboratories, the outcomes may not be directly generalizable outside UMC Utrecht. As the algorithm is up and running at UMC Utrecht, a simple experiment to test the potential performance of the app in other laboratories would be to send a series of digital slides from those laboratories to UMC Utrecht for evaluation. In this way, laboratories can easily determine whether this performance motivates them to buy the algorithm. In all laboratories using IHC up front, potential cost savings will be different. First, there may not be pathologist time savings, as pathologists would have to first look at the HE slides with the AI output, although this can be relatively fast because one only has to screen the AI annotations per slide. However, if metastases are detected, it also saves pathologists looking at the IHC slides. In contrast, the potential IHC cost savings may be even higher as more stains may be prevented, which was confirmed in our detailed calculations of potential cost savings (Methods). Importantly, if IHC stains can be omitted, this means that the diagnosis for a person can be faster as IHC staining usually takes from a few hours up to 1 day. Altogether, the cost saving incentive may also be strong for laboratories performing IHC up front.

A limitation of our study is that we did not randomize SN specimens in a case-wise manner. Switching from AI assistance to standard of care was deemed impracticable in a hectic diagnostic workflow by the participating pathologists. Furthermore, case mix variations or time trends were deemed highly unlikely to occur within the time period of inclusion^20^. Moreover, as our expert breast pathologists works according to a biweekly schedule, switching arms every 2 weeks ensured that the pathologists themselves were also randomly distributed between both arms. Nevertheless, this resulted in a significantly uneven distribution of SN metastases overall and a significantly uneven distribution of the types of these metastases (ITCs, micrometastases or macrometastases). We chose to adjust for this using a log-binomial model^21–28^, which rendered an adequately interpretable aRR (corrected for tumor size). As IHC use for detection is a common outcome (42.4%), we clearly could not interpret the adjusted odds ratio (aOR = 0.169, 95% CI: 0.022–0.797) derived from the more commonly used logistic regression model for binary data as an aRR. Nevertheless, both regression models showed a significant advantage for the use of AI assistance.

Another limitation of our study was the limited number of time measurements performed by two of the participating pathologists (mainly pathologist B) for practical reasons. Although the results showed a significant time advantage of AI assistance, we would have ideally quantified this more robustly using automatic time measurements in all cases. In this light, it is also important to mention that, if we were not to perform IHC in all morphologically negative cases, pathologists may be prompted to look (even) more diligently at the HE sections. Therefore, the time savings of AI assistance within this trial have to be interpreted with caution. Lastly, in hindsight, our sample size calculations were quite optimistic as we expected the algorithm to find all ITCs and micrometastases for which the pathologists needed IHC. This was the case for micrometastases, whereas this was not the case for ITCs. However, unassisted pathologists in the control arm also found fewer metastases without IHC than expected. In the end, our main outcome measure showed a significant advantage of AI assistance.

## Conclusion

The implementation of an AI-assisted workflow led to a significant reduction in IHC use and subsequent costs for the detection of SN metastases in persons with breast cancer, while saving pathologists time and making their work more enjoyable. Importantly, AI implementation during this trial was safe and participants were not at risk of an inferior diagnosis. Within this trial alone, an estimated 3,000 € for IHC use was saved. Depending on the current laboratory policy and the choice of a future policy regarding IHC use (that is, ‘maintaining current safety standards’ versus ‘personalized IHC use’), potential cost savings range from 1,500 to 12,500 € per 100 SNs. These cost savings are highly relevant in the current era of skyrocketing healthcare costs and limited resources. As opposed to many innovations, the implementation of AI in pathology laboratories that are fully digital may reduce healthcare costs in spite of the investments needed in AI, highlighting important benefits for pathologists and the laboratory workflow. Such tangible cost and time savings demonstrate the potential of AI implementation to keep accurate diagnostic pathology affordable and viable.

## Methods

### Study samples and pathologists

From September 2022 to May 2023, we enrolled all SN specimens from participants with biopsy-confirmed invasive breast cancer or ductal carcinoma in situ (DCIS), which were assessed by a pathologist at UMC Utrecht (the Netherlands). These specimens were derived from patients from either our academic hospital or the non-academic Alexander Monro Breast Cancer Hospital. SN specimens assessed as part of a second opinion were excluded.

Each SN specimen consisted of all unilateral SNs per participant, as they were assessed as a single set of SN slides. At grossing (by a pathologist assistant or pathologist in training), SNs were placed in one or multiple cassettes blinded to trial arm allocation. These cassettes were then formalin fixed and routinely processed into paraffin tissue blocks. Multiple small SNs may fit within one block (after color marking), while large SNs may have to be sliced and processed into several blocks. Five levels were assessed per tissue block at UMC Utrecht. Hence, the overall SN specimen may consist of one or more SNs and the total number of assessed slides for that overall SN specimen is five times the total number of SN blocks.

The group of participating pathologists consisted of all five expert breast pathologists who cover the assessment of breast specimens on the basis of a weekly schedule. These five pathologists had an age range of 33–66 years and their years of experience ranged from 1 to >30 years. The pathology laboratory at UMC Utrecht has been operating a fully digital workflow since 2015 (ref. ^29^). As such, all participating pathologists were very familiar with the digital workflow, where slides are digitized as whole-slide images (WSIs) by Hamamatsu S360 scanners at ×40 magnification and reviewed using Sectra’s PACS.

### Study design

In this single-center, two-arm interventional trial (International Standard Randomized Controlled Trial Number: 14323711), we pragmatically allocated eligible SN specimens on the basis of a biweekly time schedule to either the control arm or the intervention arm. In this way, the participating pathologists were active in both arms. Weeks of inclusion were fully completed up to the end of the trial, thereby resulting in an uneven number of cases per arm.

In the control arm, HE slides of the SN specimens were digitally assessed according to the standard clinical workflow, where IHC stains (type cytokeratin CAM 5.2) were performed in all cases where metastases were morphologically absent on HE. When metastases were morphologically identified, no confirmatory IHC stains were subsequently performed. Therefore, the reference standard (or ‘true disease status’) was based on the pathologist’s conclusion, with or without the use of IHC, as in clinical practice. When IHC was performed, this was done on a serial section kept at block cutting.

In the intervention arm, the CE-IVD-approved Metastasis Detection app (certified under IVD Regulation (IVDR), purchased from Visiopharm; intended use: assistance of pathologists) first analyzed the WSI of the SN specimens, after which one of the breast pathologists performed the first assessment of the SN specimen with the output of the algorithm available. The output was reviewed within the Visiopharm viewer app that can be called from within Sectra PACS. Again, as in the control arm, additional IHC stains were always performed when no metastases were morphologically identified at first assessment (Fig. 1).

The output of the Metastasis Detection app marks suspicious cells with red, orange or yellow outlines. As mentioned by Visiopharm in their package insert, the probability of a region being metastatic indicated by the red, orange and yellow outlines relates to the probability distribution produced by the softmax function of the neural network and should not be interpreted as confidence in the traditional sense. The app is configured to produce results at the operating points 95%, 80% and 50%, which are outlined in the colors red (high probability), orange (medium probability) and yellow (low probability), respectively. The best balance between sensitivity and specificity is found at the 95% operating point (which corresponds to the red outlines). For the largest suspicious area, a diameter is also provided by the app. An example of its output with all three outlines is shown in Fig. 1.

Pathologists could use the algorithm as pleased. However, the participating pathologists mentioned that they checked all annotations on all slides, unless obvious metastases were already detected and screening other small (usually yellow) annotations would not result in a different conclusion.

Standard Protocol Items: Recommendations for Interventional Trials (SPIRIT) AI guidelines were followed in the design of this trial^30^. Further information on research design is available in the Nature Portfolio Reporting Summary linked to this article.

### Data collection

All data were collected from the structured pathology reports and PACS and securely stored in Castor EDC^31^. In the case of a pathologic complete response after neoadjuvant therapy, tumor characteristics such as histologic subtype, histologic grade, lymphovascular invasion and receptor status were taken from the biopsy report. Additional data were collected from two surveys. The first was a survey among the participating pathologists on their user experience of the AI-assisted workflow. These questions were modified from the System Usability Scale^32^ and pathologists answered ten statements on a scale of 1 (strongly disagree) to 5 (strongly agree). To explore the potential impact of large-scale implementation of an algorithm such as the one used in this trial, a second survey was sent to all Dutch pathology laboratories to gain insight into their SN pathology workflow. Although national guidelines are in place, it is known that these guidelines are usually lagging behind evolving clinical practice and that there are differences among Dutch pathology laboratories in SN assessment (that is, the number of levels on which SNs are assessed and policy regarding IHC use)^3^.

### Study endpoints

The primary endpoint of this trial was the RR of IHC use per detected case of SN metastasis.

Secondary endpoints were divided into three categories.

1. Workflow improvements:
  - Differences between both arms in time spent per SN specimen, measured using a stopwatch by a researcher (C.v.D.) sitting next to the pathologist assessing the slides. For practical reasons, these measurements were only performed during a few weeks within the third and fourth months of the trial.
  - Difference in absolute number of IHC stains and subsequent costs (indicative cost of ~25 € per section) between both study arms, stratified for type of metastasis (ITC, micrometastasis or macrometastasis).
2. Pathologist performance in both arms:
  - Sensitivity and NPV of the pathologist on the HE slides, stratified for type of metastasis (ITC, micrometastasis or macrometastasis).
  - AI user experience (questionnaire) of the participating pathologists (Extended Data Table 1).
3. AI performance:
  Standalone performance of the algorithm was assessed by one of the researchers (C.v.D.).This assessment consisted of checking whether the annotated metastases (by the pathologist) on the HE slide or the IHC slide were also annotated by the algorithm. This outcome was binary; metastases were either annotated (regardless of color—red, orange or yellow) or not. In cases of doubt, the researcher consulted a pathologist (P.J.v.D.).
  - Retrospective standalone performance of the algorithm for cases with metastases in the control arm (sensitivity), stratified for type of metastasis (ITC, micrometastasis or macrometastasis).
  - Standalone performance (sensitivity) of the algorithm in the intervention arm.
  - Overall combined performance in both arms.

Lastly, from the obtained parameters (distribution of SN outcome, average number of tissue blocks and slides, sensitivity of AI-assisted pathologists and laboratory SN workflow), we calculated potential cost savings in different scenarios (Methods). Parameters in this file are adjustable, thereby enabling individualized calculations of potential cost savings.

### False-positive interpretations and false-positive alerts

A crucial distinction was made between false-positive interpretations by the pathologist(s) (either AI-assisted or not) and false alerts by the algorithm itself (being yellow, orange or red outlines). The first type of false positive cannot be confirmed in this study as, by design, like in clinical practice, no confirmatory stains were performed when the pathologist (either AI-assisted or not) concluded that tumor cells were present on the HE slides. However, a retrospective study by Challa et al.^33^ with the same algorithm by Visiopharm did report on this type of false-positive pathologist interpretation and showed that they are extremely rare. The authors reported similarly high rates of concordance between the ground truth and the interpretation of three subspecialized breast pathologists, either assisted by AI or not (98–100%). Moreover, false positives occurred slightly more when pathologists interpreted IHC results (two of three pathologists, 1–2 of 102 cases) versus when pathologists interpreted AI results (one of three pathologists, 1 of 102 cases). In addition, we firmly believe that no pathologist is biased toward making more cancer diagnoses (either AI-assisted or not) because these dedicated pathologists are fully aware of the diagnostic pitfalls and clinical consequences of their conclusions for patients. Therefore, although false-positive interpretations are possible in theory (not measured in this trial), they very rarely occur in daily practice and they especially do not seem to occur more when pathologists are AI assisted, indicating a potential overreliance on AI.

As for the false-positive annotations, these were quantified in the AI arm for all cases when metastases were incorrectly identified by the algorithm. For these slides, the average number of yellow, orange and red annotations per slide was counted by two of the authors (C.v.D. and N.t.H.). Regardless of their proximity, all individual annotations were counted as separate annotations.

### Ethical compliance and trial registration

As this trial investigated the effect of an intervention (AI assistance) on provider performance (pathologists’ use of IHC), the subjects were healthcare providers (pathologists) rather than the participants whose SN samples were assessed. Therefore, registration of this trial was not required according to the International Committee of Medical Journal Editors (ICMJE) guideline. Because our industrial partners expressed a wish to have the trial registered anyway, we decided to register the trial retrospectively.

Participants in this trial were not subjected to procedures and they were not required to follow any rules. Therefore, this trial was not subject to the (Dutch) Medical Research Involving Human Subjects Act (WMO); subsequently, the ethics committee (MREC NedMec) waived the need for ethical approval. Importantly, participants in this trial were not at risk of any harm. There was no risk of an inferior diagnosis (that is, missed tumor cells) as IHC stains were performed in all cases where metastases were morphologically absent at first assessment. Furthermore, the algorithm was not used independently and all cases were also analyzed by a pathologist, which further minimized the risk of a false diagnosis based on the algorithm alone. Taking the above into account and as participant data were anonymized to the researchers, the local data protection officer (DPO) and research quality coordinator (QC) also waived the need for informed consent and a data monitoring committee.

### Statistical analysis

For comparisons between both arms, parametric or non-parametric measures were used when appropriate for continuous (*t*-test or Mann–Whitney U test) and categorical variables (chi-squared test or Fisher’s exact test).

For the analysis of the primary endpoint, the proportions of IHC use in all cases of detected SN metastases were compared and aRRs were calculated using a log-binomial regression model^21–26,34^, with starting values provided by the simple approach suggested by Schwendinger et al.^27,28^ and 95% CIs calculated by bootstrapping (*n* = 1,000)^21^.

Potential confounders were identified a priori during discussions with all participating pathologists. Two factors may have a role here. First, how diligently a pathologist looks at the HE section could potentially be influenced by tumor characteristics on biopsy. However, all participating pathologists stated that they virtually always look at an SN without previously looking at any of the tumor characteristics on biopsy. In this light, it is also important to mention that the SN specimen is always assessed 1 day before the resection specimen of the tumor itself. Hence, many (if not all) tumor characteristics (either derived from the biopsy or the resection specimen) are unknown to the pathologist who assesses the SN specimen. Second, some tumor characteristics may influence the visibility of metastasized cells. Two of these potential confounding factors were identified. First, the size of the metastasis (that is, macrometastasis (≥2 mm), micrometastasis (<2 mm) or ITC) influences its visibility to a pathologist (either with or without AI assistance) and, consequently, influences the use of IHC. The same holds true for histologic subtype as, for example, lobular cancer cells are known to be more difficult to identify on the HE section.

For the secondary endpoint measurements of pathologist and AI performance, the sensitivity and the NPVs were presented as point estimates with 95% CIs calculated using the exact binomial method. Results of the questionnaire on the SN workflow of Dutch pathology laboratories were summarized as frequencies and percentages. Results of the questionnaire among participating pathologists were averaged and presented per question.

Sample size was calculated for the primary endpoint (RR of IHC use per detected case of SN metastasis). The sample size calculation was based on a retrospective analysis of 83 consecutive SN specimens from a period of 3 months at UMC Utrecht. We assumed that the AI-assisted pathologist would detect all metastases without IHC for which IHC is currently needed, which are mainly micrometastases and ITCs (~15%). Of the 83 cases, IHC was used in a total of 68 cases (0.819), mainly consisting of negative cases and 14 cases of ITCs and micrometastases. We assumed that these 14 cases would be detected by the algorithm, without the need for IHC. This resulted in a presumed proportion of IHC use in the intervention arm of 0.650 (54 of 83). This sample size calculation was, thus, built on two assumptions: a presumed similar overall distribution of negative cases and cases of ITC, micrometastases and macrometastases during the trial and a presumed proportion of IHC use in the intervention arm based on assumptions of the accuracy of the algorithm. Therefore, the sample size calculation was indirect in theory. However, it was deemed the best way to calculate clinically applicable sample sizes for this trial.

We used a one-sided significance level of 5%, as it was deemed impossible to use more IHC after AI assistance, and a power of 80%, resulting in a sample size of 166 SNs (83 per arm). As there were uncertainties on the assumption of the number of metastases that the AI-assisted pathologist would detect without IHC, we decided to include 180 SNs (90 per arm) to be on the safe side.

Data analysis was performed with IBM SPSS Statistics (version 27.0) and RStudio (version 4.2.1)^35^, with the significance level set at *P* < 0.05.

### Reporting summary

Further information on research design is available in the Nature Portfolio Reporting Summary linked to this article.

### Supplementary information

Supplementary Information Study protocol, statistical analysis plan and SPIRIT-AI checklist.

Reporting Summary

Supplementary Data 1 Explainer scenario ‘maintaining current safety standards’ and explainer scenario ‘personalized IHC use’. Parameters in this file are adjustable, thereby enabling individualized calculations of potential cost savings.

## Figures and Tables

**Extended Data Table 1:**
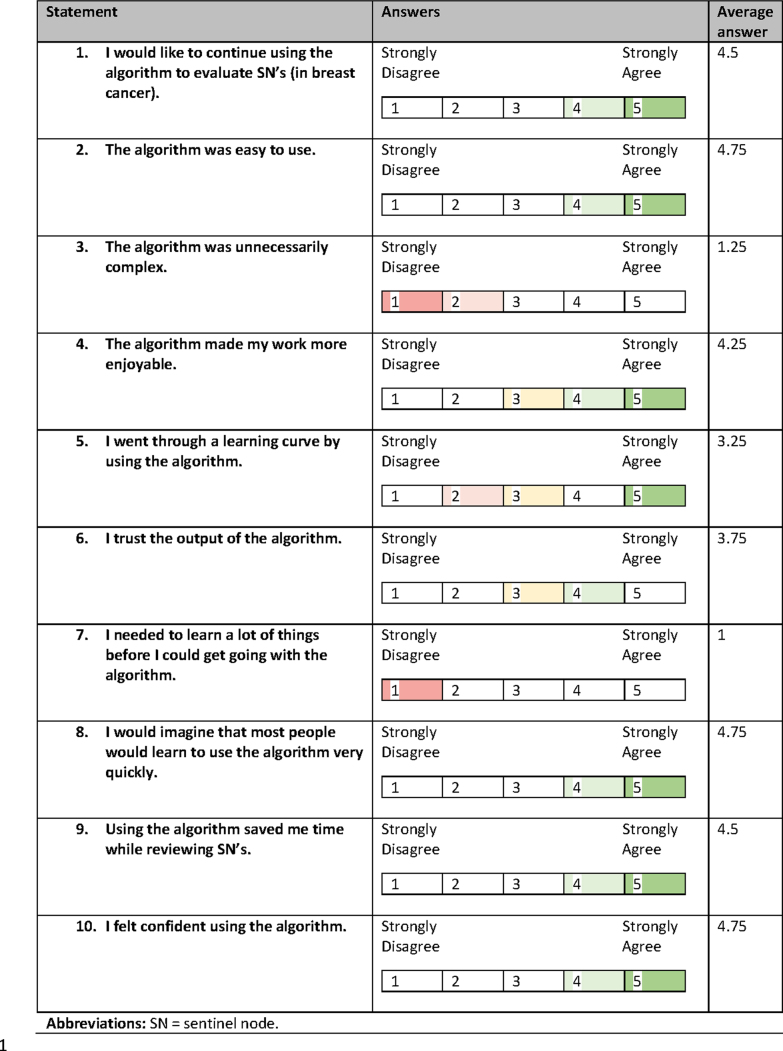
User experience survey among participating pathologists that used the algorithm (n = 4)

## Data Availability

The data within this trial were derived from the structured pathology reports and information in PACS from all persons with consecutive breast cancer or DCIS with an SN. These data were securely stored in Castor EDC^31^. All relevant data supporting the findings of this study are available within the paper and its Supplementary Information. The raw data that support the findings of this study are not openly available because of reasons of patient privacy but are available from the corresponding author upon reasonable request. Data are located in controlled access data storage at UMC Utrecht.

## Code availability

No specific code was designed for this trial. We used the CE-IVD-approved (certified under IVDR) deep-learning Metastasis Detection app by Visiopharm (Hoersholm, Denmark). This is a commercially available AI app, which was purchased from Visiopharm by our pathology department. All relevant information regarding the app can be obtained from Visiopharm (see also https://visiopharm.com/app-center/app/metastasis-detection-ai/).
