# Author Correction: Clinical implementation of artificial-intelligence-assisted detection of breast cancer metastases in sentinel lymph nodes: the CONFIDENT-B single-center, non-randomized clinical trial

**DOI:** 10.1038/s43018-024-00799-w

**Published:** 2024-07-05

**Authors:** C. van Dooijeweert, R. N. Flach, N. D. ter Hoeve, C. P. H. Vreuls, R. Goldschmeding, J. E. Freund, P. Pham, T. Q. Nguyen, E. van der Wall, G. W. J. Frederix, N. Stathonikos, P. J. van Diest

**Affiliations:** 1 Department of Pathology, University Medical Center Utrecht, Utrecht, The Netherlands; 2 Department of Medical Oncology, University Medical Center Utrecht, Utrecht, The Netherlands; 3 Department of Epidemiology and Health Economics, Julius Center for Health Sciences and Primary Care, University Medical Center Utrecht, Utrecht, The Netherlands

**Keywords:** Cancer screening, Pathology, Machine learning, Cancer imaging, Breast cancer

Correction to: *Nature Cancer* 10.1038/s43018-024-00788-z, published online 27 June 2024

In the version of the article initially published, the arrows were missing in Fig. 1 between both instances of “Pathologist” and “No tumor”. The figure has now been amended in the HTML and PDF versions of the article.

