# Supplementary material for: Clinical implementation of artificial-intelligence-assisted detection of breast cancer metastases in sentinel lymph nodes: the CONFIDENT-B single-center, non-randomized clinical trial
Source: Nat Cancer. 2024 Jun 27;5(8):1195–205. doi: 10.1038/s43018-024-00788-z (PMC11358151; doi:10.1038/s43018-024-00788-z)
Supplement: Supplementary file 1 — Study protocol, statistical analysis plan and SPIRIT-AI checklist. [file 43018_2024_788_MOESM1_ESM.pdf]

nature cancer

Supplementary information

https://doi.org/10.1038/s43018-024-00788-z

Clinical implementation of artificial-intelligence-assisted detection of breast cancer metastases in sentinel lymph nodes: the CONFIDENT-B single-center, non-randomized clinical trial

In the format provided by the authors and unedited

PROTOCOL TITLE ‘Clinical implementation of AI-assisted tumor detection in digital pathology (CONFIDENT) ’

**Short title:** CONFIDENT	
**Version**: 1	
**Date:** December 28 2021	
**Coordinating investigator/project leader**	Prof. dr. Paul van Diest
**Principal investigator (in Dutch: hoofdonderzoeker/ uitvoerder)**<br><br>**Other investigator(s)**	Prof. dr. Paul van Diest<br><br>Dr. C. van Dooijeweert<br>Drs. R. N. Flach
**Sponsor (in Dutch: verrichter/opdrachtgever)**	RvB UMC Utrecht (investigator initiated)
**Subsidising party** ***<if applicable>***	Not applicable

INTRODUCTION AND RATIONALE

Since the introduction of digital pathology, the number of studies on artificial intelligence (AI) within the field of pathology have increased rapidly.[1,2] These techniques have great potential to improve diagnostics by improving accuracy, reproducibility and speed.[2] Algorithms have been created for tumor detection, tumor grading, recognizing tumor subtypes, evaluating biomarkers and more.[1] Several of these algorithms have shown to be comparable, or even superior to pathologists.[2–8] Worldwide, a shortage of pathologists exists[9]. Combined with an increasing workload, this means that AI has great potential to alleviate the workload for pathologists.[2]

However, AI has hardly been implemented in daily pathology practice. This is mainly because most of the datasets on which the existing algorithms were trained are considered not representative of daily clinical practice data, which are subject to many more sources of variation.[2] However, AI keeps evolving and many of the above mentioned problems may have been overcome by the first FDA- and IVDR-approved algorithms, which are currently being released to the market. Lastly, there is a lack of prospective studies that pave the way for clinical implementation. As with any innovation in medicine these studies are required to address the true additional value of AI in pathology.

We an IVDR-approved algorithm for detecting lymph node metastases in breast cancer (BCa) patients. Here, the task of the pathologist is both labor-intensive and costly, due to performed immunohistochemistry (IHC) stainings in case no tumor cells are morphologically observed. However, these stainings are expensive and their costs sometimes even exceed reimbursement for the entire resection specimen. This raises the question whether artificial intelligence may be of added value to morphologically detect cancer cells without the need for IHC-use. Thereby the amount of performed stainings may be reduced, which may keep health care costs sustainable, while potentially decreasing the workload of pathologists as well.[2]

As breast cancer is the most common (non-skin) malignancy women, implementation of AI-assistance may have a great impact on diagnostic processes.

This study therefore aims to investigate the added value of AI-assistance for pathologists (augmented intelligence) in the identification of lymph node metastases in breast cancer patients.

1. OBJECTIVES

Primary Objective

To evaluate the added value of AI-assistance in detection of lymph node metastases in breast cancer in daily pathology practice, with regard to the number of spent resources (number of immunohistochemistry stains (IHC)), while maintaining diagnostic safety standards (by means of pathologist's supervision and staining in all negative cases).

Secondary Objective(s)

- To evaluate the added value of AI-assistance with regard to workflow improvements (time and number of IHC-stains and costs)
- To evaluate pathologists performance (sensitivity and negative predictive value) in both trial-arms, and AI user-experience in the intervention arm.
- To evaluate AI-performance in both arms (standalone AI-performance in the intervention arm, retrospective standalone AI-performance in the control arm, and combined overall standalone AI-performance).

2. STUDY DESIGN

The study will be a pragmatic diagnostic intervention trial, envisioned to start in April 2022 and end early 2023. The study settings are as follows:

All sentinel lymph node (SN) specimens of patients with breast cancer being assessed in the UMC Utrecht (these patients may treated either in the UMC Utrecht or the Alexander Monro hospital) will be included sequentially.

General group assignment

SN-specimens of breast cancer patients will be assigned to be assessed by a pathologist with or without AI-assistance in a pragmatic bi-weekly sequential design. This is deemed feasible as case-mix variation and time trends have not been observed previously, and as this is highly unlikely to occur within the envisioned inclusion-period of approximately six-nine months. Furthermore, specialized breast-pathologists within the UMCU work according to (bi)weekly schedules. Therefore using AI every two weeks, as opposed to switching by day, ensures that all breast pathologists are equally distributed between groups. Lastly, it would be unworkable to switch from AI-assistance to no AI-assistance on a case to case basis.

Current clinical workflow

Sentinel lymph node specimens are first assessed digitally by the pathologist, on the regular hematoxylin and eosin (H&E) staining. If the pathologist detects metastases, no additional IHC stainings will be performed. If no metastases are detected on the H&E slide by the pathologist, additional IHC stainings are performed in all cases, to ensure no metastases are being missed.

Proposed clinical workflow (see figure below)

Pathologists in the control arm will perform assessment according to the current clinical workflow. Pathologists in the AI-intervention arm will assess the HE-specimens digitally after the algorithm has assessed these. The output of the algorithm will be available to the pathologist when they assess the H&E specimen. Like in the control arm, if no metastases or tumors are detected on the H&E slide with AI assistance, additional IHC stainings are being performed in all cases, to ensure no metastases or tumors are being missed.

Therefore, the only direct benefit from the algorithm, in this first clinical introduction of AI tumor detection, will be the confirmation of metastases or tumor by the AI-assisted pathologist, in cases for which the non-AI-assisted pathologist would need IHC.

In a second phase, as the reference standard (IHC) will have been determined in all cases for which the AI-assisted pathologist concluded that no tumor or metastases were morphologically visible, we will be analyzing the safety of omitting IHC in these cases.

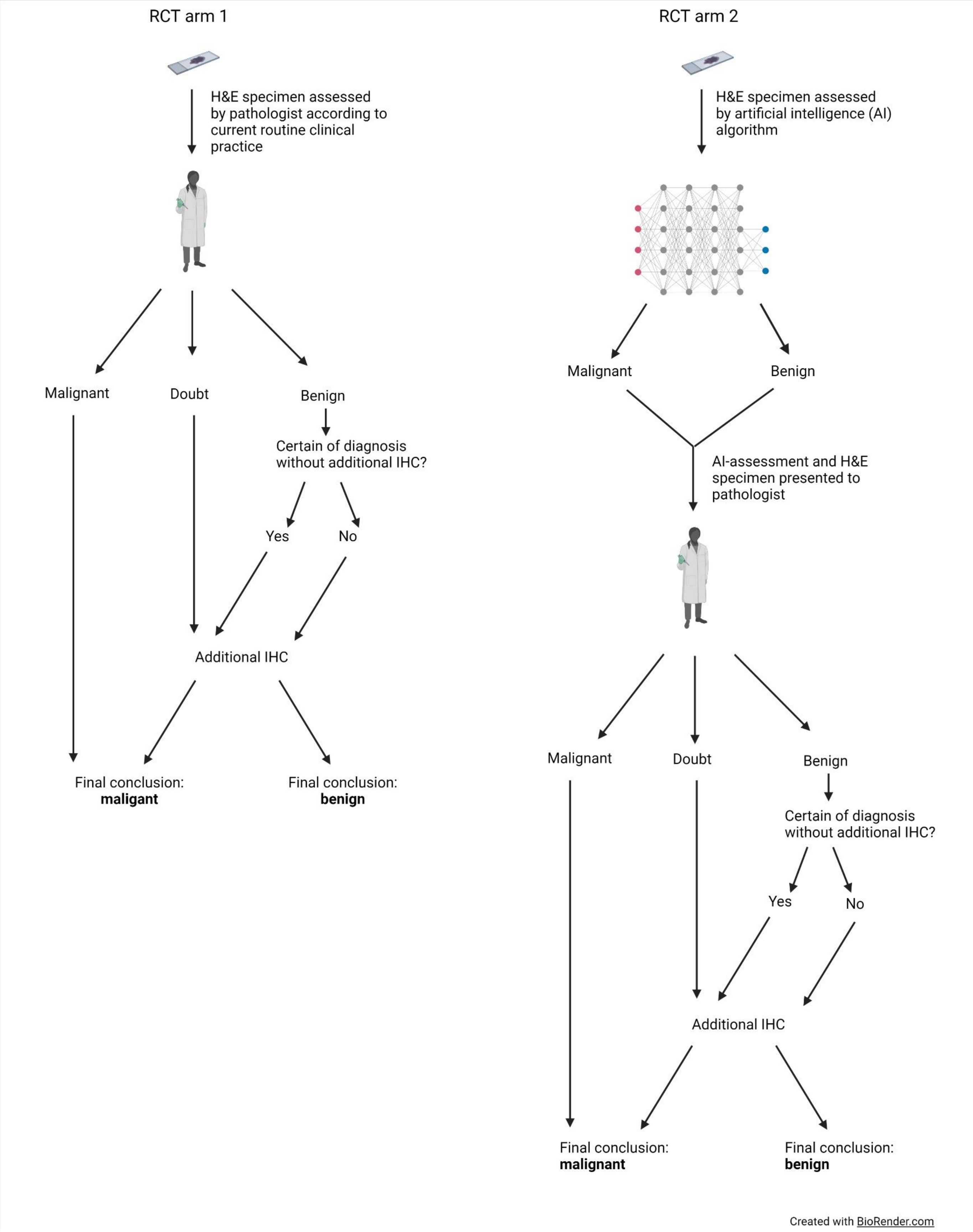
RCT arm 1
H&E specimen assessed by pathologist according to current routine clinical practice
Malignant
Doubt
Benign
Certain of diagnosis without additional IHC?
Yes
No
Additional IHC
Final conclusion: **maligant**
Final conclusion: **benign**
RCT arm 2
H&E specimen assessed by artificial intelligence (AI) algorithm
Malignant
Benign
AI-assessment and H&E specimen presented to pathologist
Malignant
Doubt
Benign
Certain of diagnosis without additional IHC?
Yes
No
Additional IHC
Final conclusion: **malignant**
Final conclusion: **benign**
Created with BioRender.com

3. STUDY POPULATION

3.1 Population (base)

All SN-specimens of women with a biopsy confirmed diagnosis of breast cancer being assessed in the UMC Utrecht , starting from (envisioned) April 2022 will be included and to have their lymph node specimen to be assessed by a pathologist with or without AI assistance.

3.2 Inclusion criteria

In order to be eligible to participate in this study, a subject must meet all of the following criteria:

- Females any age
- Breast cancer as primary malignancy
- SN-specimen assessed at the UMC Utrecht

3.3 Exclusion criteria

- Revisions from other clinics

3.4 Sample size calculation

All SN-specimens assessed within the UMC Utrecht for three consecutive months (August 1st – November 1st 2021) were analyzed. In total, 83 SN-specimens were assessed, for which in 68 cases (81.9%) IHC was performed.

In 24/83 SN-specimens metastases were detected (8 macro-metastases, 8 micro-metastases, and 8 x isolated tumor cells).

o All 8 macro-metastases were detected without IHC
o 2/8 micro-metastases were detected without IHC
o None of the 8 cases with isolated tumor cells were detected without IHC

We presume that the AI-algorithm will detect all metastases for which currently IHC is used. This means that for 14 metastases (6 micro-metastases + 8 ITC) no IHC will be used in the intervention group, whereas in the control group, IHC would be used. This leads to the following proportions per arm:

- P1 = 68/83 = 0.819
- P2 = (68-14)/83 = 0.651

A one-sided test was used, as we are only interested in a one-sided outcome, i.e. less IHC being performed in the AI-assisted arm. In addition, it is not likely that more IHC will be performed in the AI-assisted arm, as in both arms pathologists make the call whether to perform IHC. So even if the AI-algorithm would not recognize any metastases, the

pathologist would still look at the HE-slide and check whether there are no metastases (morphologically), which is exactly what the pathologists in the other arm are doing.

Calculations were performed in R by using the power.prop.test command (see below).

```
> power.prop.test(n=NULL, p1=0.819277108, p2=0.650602409, sig.level=0.05, power=0.8, alternative = "one.sided")

     Two-sample comparison of proportions power calculation

              n = 83.61041
             p1 = 0.8192771
             p2 = 0.6506024
      sig.level = 0.05
          power = 0.8
    alternative = one.sided

NOTE: n is number in *each* group
```

We decided to be conservative and include 90 SN-specimens (i.e. from 90 patients) per arm. This will take approximately 7 to 8 months.

4. INVESTIGATIONAL PRODUCT

4.1 Name and description of investigational product(s)

Visiopharm metastasis detection app (IVDR approved)

4.2 Summary of findings from non-clinical studies

Although the algorithm for detection of breast cancer metastases is IVDR approved, no clinical studies have been published so far. However, Visiopharm does provide a table on their website on a study including for 296 breast cancer cases from three clinical sites, reporting a sensitivity of 99.3% and a specificity of 100% for pathologists who are assisted by their AI-algorithm.
https://visiopharm.com/app-center/app/metastasis-detection-ai/?gclid=EAIaIQobChMIorPrmOCI9QIVBp53Ch2nGAKLEAAYASAAEgIXLPD_BwE

4.3 Summary of findings from clinical studies

No clinical studies have been performed so far, as only retrospective studies have been reported.

4.4 Summary of known and potential risks and benefits

Potential risks (zero); patients are at no risk to receive an inferior diagnosis, as the pathologists will always be the one making a definitive diagnosis. In case of doubt, IHC will always be performed.

Potential benefits: reduction of means (IHC), thereby reducing costs. Moreover, we envision improved time management, and an improved work flow for pathologists.

5. METHODS

5.1 Study parameters/endpoints

5.1.1 Main study parameter/endpoint

Relative risk of IHC-use per detected case of SN-metastases.

5.1.2 Secondary study parameters/endpoints (if applicable)

1. Workflow improvements:

- Difference in spent time per SN-specimen between both arms.
- Difference in absolute number of IHC-stains and subsequent costs between both study arms.

2. Pathologist performance

- Sensitivity and negative predictive value of the pathologists on the HE-slides, stratified for type of metastases (isolated tumor cells, micro metastases, macro metastases).
- AI-user experience (questionnaire).

3. AI-performance

- standalone AI-performance in the intervention arm
- retrospective standalone AI-performance in the control arm
- combined overall standalone AI-performance.

5.2 Study procedures

Main endpoint: See figure 1.

Secondary endpoint 1: Time is measured by stopwatch (researcher sitting next to pathologist), absolute number of stains is recorded and costs are calculated with a price of €25 per stain.

Secondary endpoint 2: Sensitivity and negative predictive value are calculated per type of metastases, AI user experience is determined by an online questionnaire.

Secondary endpoint 3: AI-performance is determined by the researcher in consultation with a pathologist in case of doubt.

5.3 Withdrawal of individual subjects

Not applicable.

Patient data will be anonymized, and data will be gathered as part of standard of care. Therefore patients cannot withdraw.

Although we use anonymous data, according to the UMC policy (and GDRP) we will

be excluding patients who object against the use of their data for research purposes (general ‘no-object’).

5.4 Replacement of individual subjects after withdrawal

Not applicable.

5.5 Follow-up of subjects withdrawn from treatment

Not applicable.

6. STATISTICAL ANALYSIS

Missing data are not expected as most parameters are obligatory used items in the synoptic pathology reports which are to be used.

6.1 Primary study parameter(s)

As the primary outcome measure is dichotomous, a simple Chi-squared test will be used to test for differences in the proportion of IHC-use in both arms. Furthermore, relative risks (of IHC-use) will be calculated.

For baseline comparisons between both arms, we will use the appropriate measures (parametric or non-parametric) for categorical (Chi-squared test/Fisher's exact) and continuous parameters (T-test, Mann-Whitney U test).

Although we do not expect differences in patient- and tumor characteristics between both groups, we will adjust for them if they are observed. We do not plan to perform the often used logistic regression model, but we will explore alternative models like Poisson regression with robust standard errors or log-binomial regression, as these will provide adjusted relative risks instead of odds ratio's provided by logistic regression. [11–13]

6.2 Secondary study parameter(s)

<u>Time needed to evaluate a biopsy or lymph node</u>
Will be compared by either a (parametric) T-test or a (non-parametric) Mann-Whitney U test, when appropriate.

<u>Satisfaction with AI-assisted work-flow (questionnaire)</u>
A quantitative analysis will be performed.

6.3 Other study parameters

<u>Number of IHC's that would not have needed to be performed after AI-implementation</u>
In the AI-arm, the number of IHC-stainings which, based on the evaluation of the AI-assisted pathologist, could have been omitted will be determined (i.e. the sensitivity of the AI-assisted pathologist will be determined).

7. ETHICAL CONSIDERATIONS

7.1 Regulation statement

The study will be conducted according to ‘gedragscode gezondheidsonderzoek’ and in accordance with the EU GDPR (General Data Protection Regulation) and Wet Medisch Wetenschappelijk Onderzoek.

7.2 Recruitment and consent

Informed consent is not required for the following reasons:

1. Standard care, i.e. assessment by a pathologist and IHC when no tumor cells are detected, is performed in all cases. Hence, there is no chance of an inferior diagnosis (missed tumor cells). Also, patients (more specifically their tissue specimens) are not subjected to any actions, nor are rules of conduct imposed on them. Therefore, overall patients just receive standard care and are at no risk. One could also think of it like performing an extra staining in routine diagnostics, for which informed consent is never required.
2. Secondly, all data will be anonymous to the researchers.

8. ADMINISTRATIVE ASPECTS AND PUBLICATION

8.1 Handling and storage of data and documents

Data will be anonymized to the researchers, who will access it in Castor, to ensure data security. Data will be kept for a period of 15 years. For further details please see the data management plan (DMP-Online).

8.2 Amendments

Amendments are changes made to the research after an ethical committee gave an advice non-WMO. Any change that may cause the investigation to fall within the scope of the WMO is submitted to the ethical committee that gave the non-WMO advice.

9. REFERENCES

1. Jiang Y, Yang M, Wang S, Li X, Sun Y. Emerging role of deep learning-based artificial intelligence in tumor pathology. *Cancer Commun*. 2020;40(4):154-166. doi:10.1002/cac2.12012
2. van der Laak J, Litjens G, Ciompi F. Deep learning in histopathology: the path to the clinic. *Nat Med*. 2021;27(5):775-784. doi:10.1038/s41591-021-01343-4
3. Bulten W, Pinckaers H, van Boven H, et al. Automated deep-learning system for Gleason grading of prostate cancer using biopsies: a diagnostic study. *Lancet Oncol*. 2020;21(2):233-241. doi:10.1016/S1470-2045(19)30739-9
4. Bejnordi BE, Veta M, Van Diest PJ, et al. Diagnostic assessment of deep learning algorithms for detection of lymph node metastases in women with breast cancer. *JAMA - J Am Med Assoc*. 2017;318(22):2199-2210. doi:10.1001/jama.2017.14585
5. Steiner DF, Nagpal K, Sayres R, et al. Evaluation of the Use of Combined Artificial Intelligence and Pathologist Assessment to Review and Grade Prostate Biopsies. *JAMA Netw Open*. 2020;3(11):1-14. doi:10.1001/jamanetworkopen.2020.23267
6. Ström P, Kartasalo K, Olsson H, et al. Artificial intelligence for diagnosis and grading of prostate cancer in biopsies: a population-based, diagnostic study. *Lancet Oncol*. 2020;21(2):222-232. doi:10.1016/S1470-2045(19)30738-7
7. Raciti P, Sue J, Ceballos R, et al. Novel artificial intelligence system increases the detection of prostate cancer in whole slide images of core needle biopsies. *Mod Pathol*. 2020;33(10):2058-2066. doi:10.1038/s41379-020-0551-y
8. Hekler A, Utikal JS, Enk AH, et al. Superior skin cancer classification by the combination of human and artificial intelligence. *Eur J Cancer*. 2019;120:114-121. doi:10.1016/j.ejca.2019.07.019
9. Robboy SJ, Weintraub S, Horvath AE, et al. Pathologist workforce in the United States: I. Development of a predictive model to examine factors influencing supply. *Arch Pathol Lab Med*. 2013;137(12):1723-1732. doi:10.5858/arpa.2013-0200-OA
10. Harrison JH, Gilbertson JR, Hanna MG, et al. Introduction to Artificial Intelligence and Machine Learning for Pathology. *Arch Pathol Lab Med*. 2021;145(10):1228-1254. doi:10.5858/arpa.2020-0541-cp
11. McNutt LA, Wu C, Xue X, Hafner JP. Estimating the relative risk in cohort studies and clinical trials of common outcomes. *Am J Epidemiol*. 2003;157(10):940-943. doi:10.1093/aje/kwg074
12. Lumley T, Kronmal R, Ma S. Relative risk regression in medical research: models, contrasts, estimators, and algorithms. . *UW Biostat Work Pap Ser 293 [http//www bepress com/uwbiostat/paper293]*. 2006;(July 2006).
13. Zou G. A Modified Poisson Regression Approach to Prospective Studies with Binary

Data. *Am J Epidemiol*. 2004;159(7):702-706. doi:10.1093/aje/kwh090

Statistical analysis plan for the CONFIDENT-B trial

Statistical tests for comparisons between arms
For comparisons between both arms, parametric or non-parametric measures were used when appropriate for continuous (T-test, Mann-Whitney U Test) and categorical variables (Chi-squared test/Fisher's exact test).

Primary endpoint analysis
For the analysis of the primary endpoint, the proportions of IHC-use in all cases of detected SN-metastases were compared, and adjusted risks were calculated using a log-binomial regression model (1-6), with starting values provided by the simple approach suggested by Schwendinger *et al* (7, 8) and 95% confidence intervals (CI) calculated by bootstrapping (n=1,000) (1).

Potential confounders were identified a-priori during discussions with all participating pathologists. Two factors may play a role here. First how diligently a pathologist looks at the HE-section could potentially be influenced by tumor characteristics on biopsy. However, all participating pathologists stated that they virtually always look at an SN without prior looking at any of the tumor characteristics on biopsy. In this light, it is also important to mention that the SN-specimen is always assessed a day before the resection specimen of the tumor itself. Hence, many (if not all) tumor characteristics (either derived from the biopsy or the resection specimen) are unknown to the pathologist who assesses the SN-specimen. Second, some tumor characteristics may influence the visibility of metastasized cells. Two of these potential confounding factors were identified. First, the size of metastases (i.e. macro-metastases (≥2mm), micro-metastases (<2mm) or ITC) influences their visibility to a pathologist (either with or without AI-assistance), and consequently influences the use of IHC. The same holds true for histologic subtype, as for example lobular cancer cells are known to be more difficult to identify on the HE-section.

Secondary endpoint analysis
For the secondary endpoint measurements of pathologist and AI-performance, sensitivity and negative predictive values (NPV) are presented as point estimates. Results of the questionnaire on the SN-workflow of Dutch pathology laboratories were summarized by frequencies and percentages. Results of the questionnaire among participating pathologists were averaged and presented per question.

Sample size calculations
Sample size calculations were based on a retrospective analysis of 83 consecutive SN-specimens from a period of three months in the UMC Utrecht. We assumed that the AI-assisted pathologist would detect all metastases without IHC for which currently IHC is needed, which are mainly micro-metastases and ITC (~15%). Of the 83 cases, IHC was used in a total of 68 cases (0.819), mainly consisting of negative cases and 14 cases of ITC and micro-metastases. We assumed that these 14 cases would be detected by the algorithm, without the need for IHC. This resulted in a presumed proportion of IHC-use in the intervention arm of 0.650 (54/83). This sample size calculation is thus built on two assumptions, being a presumed similar overall distribution of negative cases and cases of ITC, micro- and macro metastases during the trial, and a presumed proportion of IHC-use in the intervention arm based on assumptions of the accuracy of the algorithm. Therefore, the sample size calculation is in theory indirect. However, it was deemed the best way calculate clinically applicable sample sizes for this trial.

We used a one-sided significance level of 5%, as it was deemed impossible to use more IHC

after AI-assistance, and a power of 80%, resulting in a sample size of 166 SNs (83 per arm). As there are uncertainties on the assumption of the amount of metastases that the AI-assisted pathologist would detect without IHC, we decided to include 180 SNs (90 per arm) to be on the safe side.

Statistical software

Data-analysis were performed with IBM SPSS Statistics version 27.0 and RStudio version 4.2.1, with a significance level set at $p<0.05$.

References

1. McNutt LA, Wu C, Xue X, Hafner JP. Estimating the relative risk in cohort studies and clinical trials of common outcomes. Am J Epidemiol. 2003;157(10):940-3.
2. Lumley T; Kronmal RM, S. Relative Risk Regression in Medical Research: Models, Contrasts, Estimators, and Algorithms. UW Biostatistics Working Paper Series. 2006; Working Paper 293.
3. Zou G. A modified poisson regression approach to prospective studies with binary data. Am J Epidemiol. 2004;159(7):702-6.
4. Knol MJ, Duijnhoven RG, Grobbee DE, Moons KG, Groenwold RH. Potential misinterpretation of treatment effects due to use of odds ratios and logistic regression in randomized controlled trials. PloS One. 2011;6(6):e21248.
5. Mittinty MN, Lynch J. Reflection on modern methods: risk ratio regression—simple concept yet complex computation. Int J Epidemiol. 2022;52(1):309-14.
6. Knol MJ. [Down with odds ratios: risk ratios in cohort studies and randomised clinical trials]. Ned Tijdschr Geneeskd. 2012;156(28):A4775.
7. Schwendinger F, Grün B, Hornik K. A comparison of optimization solvers for log binomial regression including conic programming. Computational Statistics. 2021;36(3):1721-54.
8. Schwendinger F. Detecting separation and infinite estimates in log binomial regression 2022. Available from: https://cran.r-project.org/web/packages/detectseparation/vignettes/infinite_estimates.html [Cited: May 11, 2023]
9. R Core Team (2018). R: A language and environment for statistical computing. : R Foundation for Statistical Computing, Vienna, Austria. Available from: https://www.R-project.org/.

Table 1 SPIRIT-AI checklist

From: Guidelines for clinical trial protocols for interventions involving artificial intelligence: the SPIRIT-AI extension

Section	Item	SPIRIT 2013 item[a]	SPIRIT-AI item		Addressed on page number[b]
**Administrative information**					
**Title**	1	Descriptive title identifying the study design, population, interventions, and, if applicable, trial acronym	SPIRIT-AI 1 (i) Elaboration	Indicate that the intervention involves artificial intelligence/machine learning and specify the type of model.	Addressed on title page
			SPIRIT-AI 1 (ii) Elaboration	Specify the intended use of the AI intervention.	Addressed on title page
**Trial registration**	2a	Trial identifier and registry name. If not yet registered, name of intended registry			Addressed in Abstract and Methods section.
	2b	All items from the World Health Organization Trial Registration Dataset			
**Protocol version**	3	Date and version identifier			Uploaded with the protocol itself, version 1
**Funding**	4	Sources and types of financial, material, and other support			Addressed in the Funding section
**Roles and responsibilities**	5a	Names, affiliations, and roles of protocol contributors			See author affiliations, there are no other protocol contributors.

Section	Item	SPIRIT 2013 item[a]	SPIRIT-AI item		Addressed on page number[b]
	5b	Name and contact information for the trial sponsor			Trial sponsor is the UMC Utrecht itself (i.e. board of directors), which is mentioned in the uploaded study protocol.
	5c	Role of study sponsor and funders, if any, in study design; collection, management, analysis, and interpretation of data; writing of the report; and the decision to submit the report for publication, including whether they will have ultimate authority over any of these activities			Addressed in the Funding section, there was no role for the funder, other than providing the funds.
	5d	Composition, roles, and responsibilities of the coordinating center, steering committee, endpoint adjudication committee, data management team, and other individuals or groups overseeing the trial, if applicable (see Item 21a for data monitoring committee)			Not applicable as this was a single center study and there was no committee or steering board involved.
**Introduction**					
**Background and rationale**	6a	Description of research question and justification for undertaking the trial, including summary of relevant studies (published and unpublished) examining benefits and harms for each intervention	SPIRIT-AI 6a (i) Extension	Explain the intended use of the AI intervention in the context of the clinical pathway, including its purpose and its intended users (for example, healthcare professionals, patients, public).	This is explained in detail in the Introduction and Methods section.
			SPIRIT-AI 6a (ii) Extension	Describe any pre-existing evidence for the AI intervention.	As described in the Introduction there was no evidence rather than the 2x2 tables provided in

Section	Item	SPIRIT 2013 item[a]	SPIRIT-AI item		Addressed on page number[b]
					the insert package from Visiopharm.
	6b	Explanation for choice of comparators			Described in the methods section. Daily clinical practice, standard practice in control-arm, immunohistochemistry in alle morphologically negative cases.
**Objectives**	7	Specific objectives or hypotheses			To which extent an AI-assisted workflow can reduce immunohistochemistry (IHC) use, while maintaining safety standards of IHC when samples are morphologically negative.
**Trial design**	8	Description of trial design including type of trial (for example, parallel group, crossover, factorial, single group), allocation ratio, and framework (for example, superiority, equivalence, noninferiority, exploratory)			Trial design is described in the Methods section. Single center, pragmatic clinical trial, two-arms.
**Methods: participants, interventions and outcomes**					
**Study setting**	9	Description of study settings (for example, community clinic, academic hospital) and list of countries where data will be collected. Reference to where list of study sites can be obtained	SPIRIT-AI 9 Extension	Describe the onsite and offsite requirements needed to integrate the AI intervention into the trial setting.	Described in Methods section. Again, single center pathology, samples derived from two hospitals.

Section	Item	SPIRIT 2013 item[a]	SPIRIT-AI item		Addressed on page number[b]
**Eligibility criteria**	10	Inclusion and exclusion criteria for participants. If applicable, eligibility criteria for study centers and individuals who will perform the interventions (for example, surgeons, psychotherapists)	SPIRIT-AI 10 (i) Elaboration	State the inclusion and exclusion criteria at the level of participants.	Can be found in Methods section: all were consecutive samples, the only exclusion criterion (which did not happen once) was second opinion cases.
			SPIRIT-AI 10 (ii) Extension	State the inclusion and exclusion criteria at the level of the input data.	See above
**Interventions**	11a	Interventions for each group with sufficient detail to allow replication, including how and when they will be administered	SPIRIT-AI 11a (i) Extension	State which version of the AI algorithm will be used.	Mentioned in the reporting summary: Visiopharm Metastasis Detection, AI ID: 90159, ver. 2.0
			SPIRIT-AI 11a (ii) Extension	Specify the procedure for acquiring and selecting the input data for the AI intervention.	Whole slide images from (sentinel) lymph nodes derived from daily pathology practice.
			SPIRIT-AI 11a (iii) Extension	Specify the procedure for assessing and handling poor-quality or unavailable input data.	If that happens in practice we scan the WSI again.
			SPIRIT-AI 11a (iv) Extension	Specify whether there is human–AI interaction in the handling of the input data, and what level of expertise is required for users.	For the input data the only interaction would be to make sure the app knows on which images to run. No real expertise required, rather than

Section	Item	SPIRIT 2013 item[a]	SPIRIT-AI item		Addressed on page number[b]
					knowing which slides contain the sentinel node and run the app on those slides by clicking a few buttons (during the trial, now automated)
			SPIRIT-AI 11a (v) Extension	Specify the output of the AI intervention.	Addressed in Figure 1 and the Methods section.
			SPIRIT-AI 11a (vi) Extension	Explain the procedure for how the AI intervention's output will contribute to decision-making or other elements of clinical practice.	Extensively discussed in the Discussion section, and throughout the whole manuscript. Intended use is **assistance** of pathologist in their daily task of determining whether tumor cells are present. If not IHC is performed so there is no risk of missing metastases (other than is accepted in practice, as pathologists can miss metastases on IHC as well)
	11b	Criteria for discontinuing or modifying allocated interventions for a given trial participant (for example, drug dose change in response to harms, participant request, or improving/worsening disease)			This is not applicable.

Section	Item	SPIRIT 2013 item[a]	SPIRIT-AI item		Addressed on page number[b]
	11c	Strategies to improve adherence to intervention protocols, and any procedures for monitoring adherence (for example, drug tablet return, laboratory tests)			This is not applicable.
	11d	Relevant concomitant care and interventions that are permitted or prohibited during the trial			This is not applicable.
**Outcomes**	12	Primary, secondary, and other outcomes, including the specific measurement variable (for example, systolic blood pressure), analysis metric (for example, change from baseline, final value, time to event), method of aggregation (for example, median, proportion), and time point for each outcome. Explanation of the clinical relevance of chosen efficacy and harm outcomes is strongly recommended			All explained in the Methods section.
**Participant timeline**	13	Time schedule of enrollment, interventions (including any run-ins and washouts), assessments, and visits for participants. A schematic diagram is highly recommended (Fig. 1)			This is not applicable, sentinel nodes are assessed at a single timepoint.
**Sample size**	14	Estimated number of participants needed to achieve study objectives and how it was determined, including clinical and statistical assumptions supporting any sample size calculations			All explained in the Methods section (including the assumptions), n = 90 per arm.
**Recruitment**	15	Strategies for achieving adequate participant enrollment to reach target sample size			This is not applicable as patients were not enrolled.
**Methods: assignment of interventions (for controlled trials)**					
**Sequence generation**	16a	Method of generating the allocation sequence (for example, computer-generated random numbers), and list of any			This is not applicable as we allocated

Section	Item	SPIRIT 2013 item[a]	SPIRIT-AI item		Addressed on page number[b]
		factors for stratification. To reduce predictability of a random sequence, details of any planned restriction (for example, blocking) should be provided in a separate document that is unavailable to those who enroll participants or assign interventions			pragmatically every two weeks, which we explain in the Abstract and Methods section as well.
**Allocation concealment mechanism**	16b	Mechanism of implementing the allocation sequence (for example, central telephone; sequentially numbered, opaque, sealed envelopes), describing any steps to conceal the sequence until interventions are assigned			See above, pragmatic, there was no allocation concealment.
**Implementation**	16c	Who will generate the allocation sequence, who will enroll participants, and who will assign participants to interventions			See above, this was not applicable.
**Blinding (masking)**	17a	Who will be blinded after assignment to interventions (for example, trial participants, care providers, outcome assessors, data analysts), and how			Not applicable, there was no blinding.
	17b	If blinded, circumstances under which unblinding is permissible, and procedure for revealing a participant's allocated intervention during the trial			
**Methods: data collection, management and analysis**					
**Data collection methods**	18a	Plans for assessment and collection of outcome, baseline, and other trial data, including any related processes to promote data quality (for example, duplicate measurements, training of assessors) and a description of study instruments (for example, questionnaires, laboratory tests) along with their reliability and validity, if known. Reference to where data collection forms can be found, if not in the protocol			These can be found in the Study protocol, reporting summary and in our baseline Table 1.

Section	Item	SPIRIT 2013 item[a]	SPIRIT-AI item		Addressed on page number[b]
**Data management**	18b	Plans to promote participant retention and complete follow-up, including list of any outcome data to be collected for participants who discontinue or deviate from intervention protocols			Not applicable, patients' samples were included, not patients themselves.
	19	Plans for data entry, coding, security, and storage, including any related processes to promote data quality (for example, double data entry; range checks for data values). Reference to where details of data management procedures can be found, if not in the protocol			Can be found in the Study protocol and Methods section. Data were securely stored in Castor EDC.
**Statistical methods**	20a	Statistical methods for analyzing primary and secondary outcomes. Reference to where other details of the statistical analysis plan can be found, if not in the protocol			Can be found in the Study protocol and Methods section, also in the statistical analysis plan.
	20b	Methods for any additional analyses (for example, subgroup and adjusted analyses)			Can be found in the Study protocol and Methods section, also in the statistical analysis plan.
	20c	Definition of analysis population relating to protocol non-adherence (for example, as randomized analysis), and any statistical methods to handle missing data (for example, multiple imputation)			Not applicable.
**Methods: monitoring**					
**Data monitoring**	21a	Composition of data monitoring committee (DMC); summary of its role and reporting structure; statement of whether it is independent from the sponsor and competing interests; and reference to where further details about its			Not applicable.

Section	Item	SPIRIT 2013 item[a]	SPIRIT-AI item		Addressed on page number[b]
		charter can be found, if not in the protocol. Alternatively, an explanation of why a DMC is not needed			
	21b	Description of any interim analyses and stopping guidelines, including who will have access to these interim results and make the final decision to terminate the trial			Not applicable.
**Harms**	22	Plans for collecting, assessing, reporting, and managing solicited and spontaneously reported adverse events and other unintended effects of trial interventions or trial conduct	SPIRIT-AI 22 Extension	Specify any plans to identify and analyze performance errors. If there are no plans for this, justify why not.	Not applicable.
**Auditing**	23	Frequency and procedures for auditing trial conduct, if any, and whether the process will be independent from investigators and the sponsor			Not applicable.
**Ethics and dissemination**					
**Research ethics approval**	24	Plans for seeking research ethics committee/institutional review board (REC/IRB) approval			We have a waiver, see Methods section.
**Protocol amendments**	25	Plans for communicating important protocol modifications (for example, changes to eligibility criteria, outcomes, analyses) to relevant parties (for example, investigators, REC/IRBs, trial participants, trial registries, journals, regulators)			Not applicable.
**Consent or ascent**	26a	Who will obtain informed consent or assent from potential trial participants or authorized surrogates, and how (see Item 32)			Not applicable.
	26b	Additional consent provisions for collection and use of participant data and biological specimens in ancillary studies, if applicable			Not applicable.

Section	Item	SPIRIT 2013 item[a]	SPIRIT-AI item		Addressed on page number[b]
**Confidentiality**	27	How personal information about potential and enrolled participants will be collected, shared, and maintained in order to protect confidentiality before, during, and after the trial			See Methods section. Researcher had no excess to patient data.
**Declaration of interests**	28	Financial and other competing interests for principal investigators for the overall trial and each study site			There were no competing interest and the paper includes a competing interest statement.
**Access to data**	29	Statement of who will have access to the final trial dataset, and disclosure of contractual agreements that limit such access for investigators	SPIRIT-AI 29 Extension	State whether and how the AI intervention and/or its code can be accessed, including any restrictions to access or re-use.	Can be found in our Code availability statement. App can be purchased from Visiopharm.<br><br>Access to final trial dataset: the authors of this paper.
**Ancillary and post-trial care**	30	Provisions, if any, for ancillary and post-trial care, and for compensation to those who suffer harm from trial participation			Not applicable.
**Dissemination policy**	31a	Plans for investigators and sponsor to communicate trial results to participants, healthcare professionals, the public, and other relevant groups (for example, via publication, reporting in results databases, or other data sharing arrangements), including any publication restrictions			Results were already discussed at the San Antonio Breast Cancer Symposium 2023 and several local conferences in the Netherlands. There are no current plans except for a press release from the hospital

Section	Item	SPIRIT 2013 item[a]	SPIRIT-AI item		Addressed on page number[b]
					when our study has been published in Nature Cancer. There are no publication restrictions.
	31b	Authorship eligibility guidelines and any intended use of professional writers			All authors have contributed and have therefore been included.
	31c	Plans, if any, for granting public access to the full protocol, participant-level dataset, and statistical code			Not applicable, see also data availability statement.
**Appendices**					
**Informed consent materials**	32	Model consent form and other related documentation given to participants and authorized surrogates			Not applicable.
**Biological specimens**	33	Plans for collection, laboratory evaluation, and storage of biological specimens for genetic or molecular analysis in the current trial and for future use in ancillary studies, if applicable			Not applicable.

1. [a]It is strongly recommended that this checklist be read in conjunction with the SPIRIT 2013 Explanation & Elaboration for important clarification on the items.
2. [b]Indicates page numbers to be completed by authors during protocol development.
