# Supplementary material for: Clinical implementation of artificial-intelligence-assisted detection of breast cancer metastases in sentinel lymph nodes: the CONFIDENT-B single-center, non-randomized clinical trial
Source: Nat Cancer. 2024 Jun 27;5(8):1195–205. doi: 10.1038/s43018-024-00788-z (PMC11358151; doi:10.1038/s43018-024-00788-z)
Supplement: Supplementary file 2 — Reporting Summary [file 43018_2024_788_MOESM2_ESM.pdf]

nature portfolio

Corresponding author(s): Dr. C. van Dooijeweert

Last updated by author(s): Apr 18, 2024

Reporting Summary

Nature Portfolio wishes to improve the reproducibility of the work that we publish. This form provides structure for consistency and transparency in reporting. For further information on Nature Portfolio policies, see our Editorial Policies and the Editorial Policy Checklist.

Statistics

For all statistical analyses, confirm that the following items are present in the figure legend, table legend, main text, or Methods section.

n/a	Confirmed	
☐	☒	The exact sample size (*n*) for each experimental group/condition, given as a discrete number and unit of measurement
☐	☒	A statement on whether measurements were taken from distinct samples or whether the same sample was measured repeatedly
☐	☒	The statistical test(s) used AND whether they are one- or two-sided<br>*Only common tests should be described solely by name; describe more complex techniques in the Methods section.*
☐	☒	A description of all covariates tested
☒	☐	A description of any assumptions or corrections, such as tests of normality and adjustment for multiple comparisons
☐	☒	A full description of the statistical parameters including central tendency (e.g. means) or other basic estimates (e.g. regression coefficient) AND variation (e.g. standard deviation) or associated estimates of uncertainty (e.g. confidence intervals)
☐	☒	For null hypothesis testing, the test statistic (e.g. *F*, *t*, *r*) with confidence intervals, effect sizes, degrees of freedom and *P* value noted<br>*Give P values as exact values whenever suitable.*
☒	☐	For Bayesian analysis, information on the choice of priors and Markov chain Monte Carlo settings
☒	☐	For hierarchical and complex designs, identification of the appropriate level for tests and full reporting of outcomes
☒	☐	Estimates of effect sizes (e.g. Cohen's *d*, Pearson's *r*), indicating how they were calculated

Our web collection on statistics for biologists contains articles on many of the points above.

Software and code

Policy information about availability of computer code

Data collection	Collection of data from structured pathology reports and PACS, secured storage in Castor EDC.
Data analysis	Data-analysis were performed with IBM SPSS Statistics version 27.0 and RStudio version 4.2.1<br>Algorithm: Visiopharm Metastasis Detection, AI ID: 90159, ver. 2.0

For manuscripts utilizing custom algorithms or software that are central to the research but not yet described in published literature, software must be made available to editors and reviewers. We strongly encourage code deposition in a community repository (e.g. GitHub). See the Nature Portfolio guidelines for submitting code & software for further information.

Data

Policy information about availability of data

All manuscripts must include a data availability statement. This statement should provide the following information, where applicable:

- Accession codes, unique identifiers, or web links for publicly available datasets
- A description of any restrictions on data availability
- For clinical datasets or third party data, please ensure that the statement adheres to our policy

The data within this trial were derived from the structured pathology reports and information in PACS from all consecutive breast cancer or DCIS patients with an SN. These data were securely stored in Castor EDC (17). All relevant data supporting the findings of this study are available within the paper and its Supplementary

Information. The raw data that support the findings of this study are not openly available due to reasons of patient privacy but are available from the corresponding author upon reasonable request. Data are located in controlled access data storage at University Medical Centre Utrecht.

Research involving human participants, their data, or biological material

Policy information about studies with human participants or human data. See also policy information about sex, gender (identity/presentation), and sexual orientation and race, ethnicity and racism.

Reporting on sex and gender	Breast cancer is not restricted, but obviously much more frequent in females. In this study, we included only one male with breast cancer, who has had his sentinel node assessed in the UMCU. Sex was reported according to the electronic patient records.
Reporting on race, ethnicity, or other socially relevant groupings	We do not report on race, ethnicity, or other socially relevant groups.<br>We did also not record any data on this level, as we do not see the relevance of this with regard to our study.
Population characteristics	Population characteristics that were recorded were: age, hospital of origin (either UMCU or Monro), previous therapy (e.g neoadjuvant therapy) and breast tumor characteristics (e.g. (y)pT-stage, histologic subtype, histologic grade, lymphovascular invasion, ER-status, PR-status, HER2-receptor status) .
Recruitment	See also manuscript itself. Patients were not recruited in this trial, which investigates the effect of an intervention (AI-assistance) on provider-performance (pathologists' use of IHC). Therefore the actual subjects are health care providers (pathologists) rather than the patients whose sentinel node samples were assessed. Furthermore, patients in this trial were not subjected to procedures and they were not required to follow any rules. Therefore this trial is not subject to the (Dutch) Medical Research Involving Human Subjects Act (WMO) and subsequently, the ethics committee (MREC NedMec) waived the need for ethical approval. Importantly, patients in this trial were never at risk of any harm. There was no risk of an inferior diagnosis (i.e. missed tumor cells) as IHC-stains were performed in all cases where metastases were morphologically absent at first assessment. Furthermore, the algorithm was never used independently and all cases were also analyzed by a pathologist, which further minimized the risk of a false diagnosis based on the algorithm alone. Taking the above into account, and as patient data were anonymized to the researchers, the local data protection officer (DPO) and research quality coordinator (QC) also waived the need for informed consent and a data monitoring committee.
Ethics oversight	MREC NedMec (https://nedmec.nl/en)

Note that full information on the approval of the study protocol must also be provided in the manuscript.

Field-specific reporting

Please select the one below that is the best fit for your research. If you are not sure, read the appropriate sections before making your selection.

☒ Life sciences ☐ Behavioural & social sciences ☐ Ecological, evolutionary & environmental sciences

For a reference copy of the document with all sections, see nature.com/documents/nr-reporting-summary-flat.pdf

Life sciences study design

All studies must disclose on these points even when the disclosure is negative.

Sample size	See also manuscript.<br>Sample size calculations were based on a retrospective analysis of 83 consecutive SN-specimens from a period of three months in the UMC Utrecht. We assumed that the AI-assisted pathologist would detect all metastases without IHC for which currently IHC is needed, which are mainly micro-metastases and ITC (~15%). Of the 83 cases, IHC was used in a total of 68 cases (0.819), mainly consisting of negative cases and 14 cases of ITC and micro-metastases. We assumed that these 14 cases would be detected by the algorithm, without the need for IHC. This resulted in a presumed proportion of IHC-use in the intervention arm of 0.650 (54/83). This sample size calculation is thus built on two assumptions, being a presumed similar overall distribution of negative cases and cases of ITC, micro- and macro metastases during the trial, and a presumed proportion of IHC-use in the intervention arm based on assumptions of the accuracy of the algorithm. Therefore, the sample size calculation is in theory indirect. However, it was deemed the best way calculate clinically applicable sample sizes for this trial. We used a one-sided significance level of 5%, as it was deemed impossible to use more IHC after AI-assistance, and a power of 80%, resulting in a sample size of 166 SNs (83 per arm). As there are uncertainties on the assumption of the amount of metastases that the AI-assisted pathologist would detect without IHC, we decided to include 180 SNs (90 per arm) to be on the safe side.
Data exclusions	We did not exclude any data. None of the included samples met our exclusion criteria.
Replication	We did not replicate anything in this study (which was a trial in daily clinical practice, not experiments), therefore this is not applicable.
Randomization	See also manuscript.<br>Allocation was not random. We allocated eligible SN-specimens, based on a bi-weekly time schedule, to either the control-arm or the intervention-arm. Covariates were controlled for by using a log-binomial regression model, with starting values provided by the simple approach suggested by Schwendinger et al, and 95% confidence intervals (CI) calculated by bootstrapping (n=1,000).

Blinding

Investigators were not blinded. This was not possible (investigators needed to check AI-output), but is also not relevant to our study as there are no soft outcome measures. Either immunohistochemistry stains were used or not.

Reporting for specific materials, systems and methods

We require information from authors about some types of materials, experimental systems and methods used in many studies. Here, indicate whether each material, system or method listed is relevant to your study. If you are not sure if a list item applies to your research, read the appropriate section before selecting a response.

Materials & experimental systems

n/a	Involved in the study	
☒	☐	Antibodies
☒	☐	Eukaryotic cell lines
☒	☐	Palaeontology and archaeology
☒	☐	Animals and other organisms
☐	☒	Clinical data
☒	☐	Dual use research of concern
☒	☐	Plants

Methods

n/a	Involved in the study	
☒	☐	ChIP-seq
☒	☐	Flow cytometry
☒	☐	MRI-based neuroimaging

Clinical data

Policy information about clinical studies

All manuscripts should comply with the ICMJE guidelines for publication of clinical research and a completed CONSORT checklist must be included with all submissions.

Clinical trial registration

ISRCTN: 14323711

Study protocol

The study protocol is uploaded during submission. A paper on the trial protocol was published in BMJ Open: https://bmjopen.bmj.com/content/13/6/e067437.long

Data collection

See also manuscript.
All data were collected from the structured pathology reports and PACS, and securely stored in Castor EDC. In case of a pathologic complete response after neoadjuvant therapy, tumor characteristics like histologic subtype, histologic grade, lymphovascular invasion and receptor status were taken from the biopsy report. Additional data were collected from two surveys. The first was a survey among the participating pathologists on their user-experience of the AI-assisted workflow. These questions were modified from the System Usability Scale and pathologists answered ten statements on a scale of 1 (strongly disagree) to 5 (strongly agree). To explore the potential impact of large-scale implementation an algorithm such as the one used in this trial, a second survey was sent to all Dutch pathology laboratories to gain insight in their SN pathology workflow.
Data were collected from September 2022 to May 2023.

Outcomes

See also manuscript, study protocol, statistical analysis plan.

The primary endpoint of this trial was the relative risk of IHC-use per detected case of SN-metastases.

Secondary endpoints were divided into three categories.
1. Workflow improvements:
a. Differences between both arms in time spent per SN-specimen, measured by stopwatch by a researcher (CvD), sitting next to the pathologist assessing the slides. For practical reasons these measurements were only performed during a few weeks within the third and fourth month of the trial.
b. Difference in absolute number of IHC-stains and subsequent costs (indicative costs ~€25 per section) between both study-arms, stratified for type of metastases (ITC, micro- or macro-metastases).

2. Pathologists performance in both arms:
a. Sensitivity and negative predictive value of the pathologist on the HE-slides, stratified for type of metastases (ITC, micro- or macro-metastases).
b. AI user-experience (questionnaire) of the participating pathologists (supplementary table 1).

3. AI-performance:
a. Retrospective standalone performance of the algorithm on the cases with metastases in the control arm (sensitivity), stratified for type of metastases (ITC, micro- or macro-metastases). Here, the AI-output was assessed by a researcher (CvD), in consultation with a pathologist (PvD), in case of doubt.
b. Standalone performance (sensitivity) of AI in the intervention-arm.
c. Overall combined AI-performance in both arms.

Lastly, from the obtained parameters (distribution of SN-outcome, average number of tissue-blocks and slides, sensitivity of AI-assisted pathologists, and laboratory SN-workflow), we calculated potential cost savings in different scenarios (Supplementary file 1). Parameters in this file are adjustable, thereby enabling individualized cost-saving calculations.
